# What is reproductive isolation?

**DOI:** 10.1111/jeb.14005

**Published:** 2022-09-05

**Authors:** Anja M. Westram, Sean Stankowski, Parvathy Surendranadh, Nick Barton

**Affiliations:** ^1^ IST Austria Klosterneuburg Austria; ^2^ Faculty of Biosciences and Aquaculture Nord University Bodø Norway

**Keywords:** adaptation, genomics, natural selection, population genetics, speciation, theory

## Abstract

Reproductive isolation (RI) is a core concept in evolutionary biology. It has been the central focus of speciation research since the modern synthesis and is the basis by which biological species are defined. Despite this, the term is used in seemingly different ways, and attempts to quantify RI have used very different approaches. After showing that the field lacks a clear definition of the term, we attempt to clarify key issues, including what RI is, how it can be quantified in principle, and how it can be measured in practice. Following other definitions with a genetic focus, we propose that RI is a quantitative measure of the effect that genetic differences between populations have on gene flow. Specifically, RI compares the flow of neutral alleles in the presence of these genetic differences to the flow without any such differences. RI is thus greater than zero when genetic differences between populations reduce the flow of neutral alleles between populations. We show how RI can be quantified in a range of scenarios. A key conclusion is that RI depends strongly on circumstances—including the spatial, temporal and genomic context—making it difficult to compare across systems. After reviewing methods for estimating RI from data, we conclude that it is difficult to measure in practice. We discuss our findings in light of the goals of speciation research and encourage the use of methods for estimating RI that integrate organismal and genetic approaches.

## Graphical abstract

Reproductive isolation (RI) is a core concept in evolutionary biology and the basis by which biological species are defined. Despite this, the term is used in different ways and efforts to quantify RI from data have used vastly different approaches. In this paper, we attempt to clarify key issues about RI, including what it is, how it can be quantified in principle, and how it can be measured in practice.

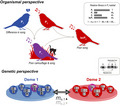

## 1 INTRODUCTION

A biological species is defined as a group of interbreeding natural populations that are reproductively isolated from other such groups (Coyne & Orr, 2004; Dobzhansky, 1937; Mayr, 1942). The notion of reproductive isolation (RI) is thus central to understanding species and speciation. But what, exactly, do we mean by ‘reproductive isolation’? Despite being deeply embedded in the language of speciation, the term is used in seemingly different ways, usually without a precise definition; attempts to quantify it have used very different approaches that measure different things. Connecting different perspectives and approaching a general definition is important for our conceptual understanding of speciation and for efforts to quantify RI empirically. The main aim of this article is to contribute to progress in this respect.

In Box 1, we briefly summarize the history of the term and present the results of a recent survey on RI among evolutionary biologists working on speciation. Both the survey and the historical overview suggest that researchers tend to focus on different aspects when they explain what RI means for them, mainly falling into two groups: a reduction in the production or fitness of hybrids (‘organismal focus’) or a reduction in gene flow (‘genetic focus’).

BOX 1 **Origin and meaning of ‘reproductive isolation’**

The term ‘reproductive isolation’ first appeared in the 1930s (Emerson, 1935), but the idea of species as reproductive communities can be traced back to Linnaeus’ emphasis on reproductive organs in taxonomic classification. Even during Darwin's time, some biologists argued that species should be distinguished from races by their inability to produce fertile offspring (Huxley, 1860). For example, Wallace (1865) stated that ‘species are merely those strongly marked races or local forms which, when in contact, do not intermix, and when inhabiting distinct areas are generally believed… to be incapable of producing fertile hybrid offspring’. Around the turn of the century, Poulton (1904) laid out a verbal theory for how interspecific sterility might evolve through the cessation of interbreeding between groups (i.e. ‘asyngamy’) owing to geographic isolation, mechanical incompatibilities, or preferential mating. As highlighted by Mallet (2004a, 2004b), this largely overlooked work laid the foundation for modern speciation research, ultimately leading to the widespread focus on RI that emerged in the mid to late 20th century (Figure B1.1A,B).

Although RI became central to the work of Mayr and Dobzhansky in the 1940s, clear definitions did not emerge until the 1950s. Dobzhansky (1951) stated that RI exists between populations when ‘the gene exchange between species is restricted or suppressed owing to genotypically conditioned differences between their populations’. He also coined the term ‘isolating mechanism’ to refer to the properties of organisms that may cause RI (1937, 1951). Mayr (1959) stated that reproductive isolation is ‘…what we might call the protective devices of a well‐integrated and harmoniously coadapted gene pool against pollution by other gene pools’. While very similar, these definitions seem to differ in regard to the precise meaning of the term. In his writing, Mayr (1942, 1959, 1963) tended to emphasize the organismal traits that restrict reproduction (and thus gene flow) between groups of organisms, whereas Dobzhansky emphasized that RI was the reduction in gene flow itself.

Focussing on other influential papers in speciation research, we found that later definitions, discussions and studies of RI varied similarly in whether they emphasize patterns of reproduction between organisms (organismal focus) or levels of gene flow between populations (genetic focus) (Table S1). However, despite being widely used in the literature, RI is usually not specifically defined, making it difficult to gauge how widespread these different views are. To address this, we turned to a recently published survey to gain additional insight (Stankowski & Ravinet, 2021b). Survey question 12 asked: ‘In a sentence or two, what is reproductive isolation?’. The answers from 231 speciation researchers (Table S2) were variable, but we could classify most based on whether they mentioned (i) patterns of interbreeding, (ii) levels of gene flow, (iii) the ability of populations to remain distinct, or some combination of the three (Figure B1.1C). Forty‐two per cent of answers had a purely organismal focus, mentioning only patterns of interbreeding (Figure B1.1D). Answers focussing only on the levels of gene flow were the second most common, accounting for 30%. Answers focussing exclusively on the distinctness of populations were uncommon, accounting for roughly 6% of the total. Seventeen per cent of the answers mentioned both an organismal and genetic perspective, though varied depending on whether RI was a reduction in interbreeding (which causes a reduction in gene flow), or a reduction in gene flow (caused by a reduction in mating). These results suggest that speciation researchers are divided on exactly what RI is, highlighting the need for clarification.

**Figure:**
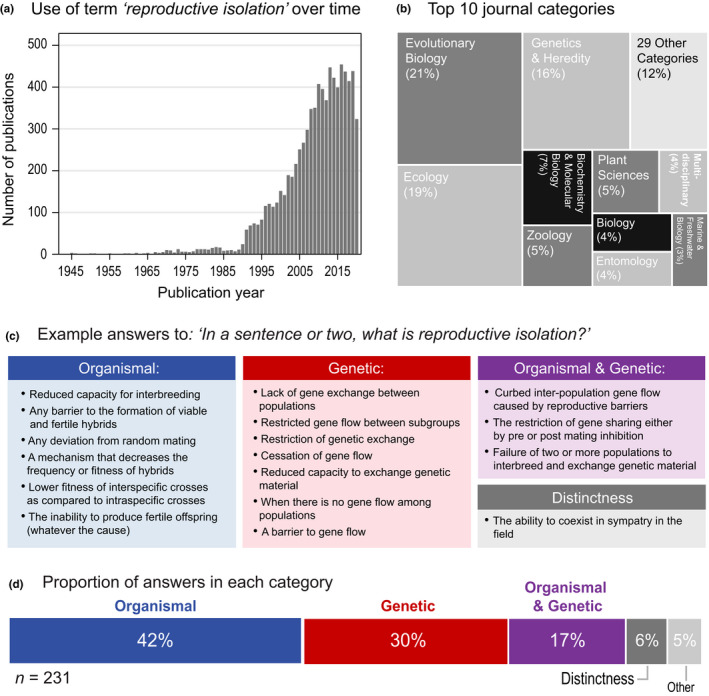
**FIGURE B1.1** Use of RI in the literature and insights into its meaning from an online survey. (a) Number of papers using the term ‘reproductive isolation’ in their title, abstract or keywords and (b) the top 10 journal categories in which the term RI is used, both according to ISI web of Science as of September 23, 2021. (c) Example answers to the question: ‘In a sentence or two, what is reproductive isolation?’, from the speciation survey, classified as described in the text. (d) The percentage of answers classified into each category. See Table S2 for the full set of answers and methodological details.

Importantly, despite these differences in focus, these perspectives of RI are not contradictory or mutually exclusive: clearly, gene flow is reduced *because* there is a reduction in the production and fitness of hybrids. Similarly, many proponents of the organismal focus emphasize that the reduced production of hybrids is relevant because it restricts gene flow and leads to the formation of genetically distinct clusters (e.g. Sobel & Chen, 2014).

**FIGURE 1:**
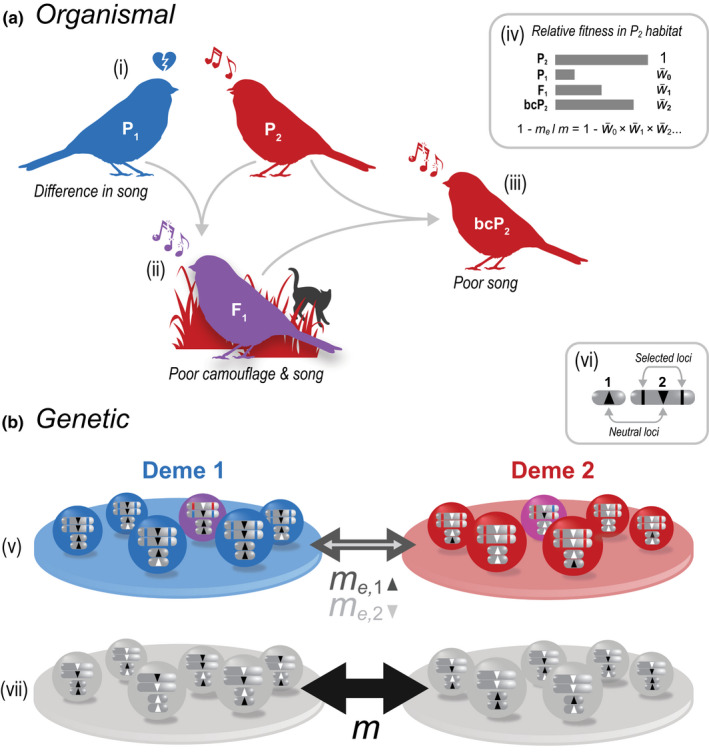
Different but complementary perspectives of reproductive isolation. (a) The ‘organismal’ perspective of RI tends to focus on the reduction in successful interbreeding between taxa. In this example, two bird populations (P_1_ and P_2_) have diverged for colour and song. Reduced attractiveness and camouflage cause immigrants, F_1_ *s*, and backcrosses (bc) to have lower mean fitness relative to the resident type (i–iii). (iv) Example relative fitnesses are given for a P_1_ immigrant (${\bar{W}}_{0}$), F_1_ (${\bar{W}}_{1}$) and P_2_ backcross (${\bar{W}}_{2}$), relative to a resident P_2_ individual. In a two‐deme model with low migration, the gene flow for an unlinked neutral locus relative to that expected without any barriers to gene flow ($m_{e}/m$) is the product of the mean fitnesses of successive hybrid classes (${\bar{W}}_{0}{\bar{W}}_{1}{\bar{W}}_{2}\ldots$). (b) The genetic perspective tends to focus on the reduction in gene flow between populations due to selection acting on genetic differences. This is illustrated for a two‐deme model with a genetic barrier (v), contrasted with a neutral scenario with no barrier (vii). In both scenarios, diploid individuals carry *n* = 2 chromosomes. (vi) Both chromosomes (1 and 2) carry a neutral locus (up and down facing triangles), each with two alternative alleles (black and white). In the barrier scenario (v), the neutral locus on chromosome 2 is flanked by a pair of loci that affect fitness; blue and red alleles at these loci maximize fitness in demes 1 and 2, respectively, but severely reduce fitness in the other deme. Although individuals migrate between demes at the same rate in both scenarios, the rate of gene flow at neutral loci—which is indicated by the width of the arrows and evident from the amount of neutral allele sharing between the demes—is lower than expected in the barrier scenario due to their association with selected loci. Note that this ‘effective’ migration rate (*m*_e_) differs between the two neutral loci, and is more strongly reduced for neutral alleles linked to the selected loci

Thus, while different perspectives highlight different aspects of RI, they are related to one another and describe the same situation: Reproductive isolation refers to a scenario involving a pair of populations; genetic differences between them lead to a reduction in hybrid formation or fitness (e.g. different adaptations, different mating preferences, or intrinsic incompatibilities), which in turn restricts gene flow (Figure 1).

Despite this common conceptual understanding of RI, there is much need for clarification, which we attempt in this article. The main points that need to be addressed include:

1. What, precisely, is reproductive isolation, and how do we unify the organismal and genetic perspective in the definition?
2. RI as verbally explained above, and as often used in the literature, refers to a feature of pairs of populations or species, rather than a quantity. In the survey (Box 1), almost none of the respondents described RI as a quantitative measure. RI was typically either described as a qualitative feature or as the complete absence of hybridization / gene flow. On the other hand, many studies use the term in a quantitative sense, e.g. comparing the level of RI between different taxon pairs. To compare different barriers to gene flow or different study systems, to study speciation over time, and to study genomic patterns, a quantitative definition of RI is necessary (e.g. Stankowski & Ravinet, 2021a). There is an understanding in the literature that RI should reflect the range from gene flow unrestricted by genetic differences at one extreme, to the absence of fertile hybrids and gene flow at the other extreme. Yet, how to quantify RI between these two extremes is unclear.
3. Divergence occurs in situations often much more complex than depicted in Figure 1. For example, divergence often happens in continuous space, making a two‐deme model unrealistic. How do we define RI in continuous or complex space, and what are the entities (i.e. populations) between which we aim to measure it in the first place? And how do we integrate the fact that RI also varies over time (e.g. when two populations have come into secondary contact after divergence in allopatry)?
4. To be useful empirically, we must be able to estimate RI from field or experimental data. There are several very different approaches to measuring RI in the literature. Importantly, when trying to quantify RI, the precise focus on organismal vs. genetic aspects of RI becomes relevant, as these are associated with very different methods (e.g. using lab crosses vs. sequencing data). Approaches also differ in whether there is a single estimate for a given population pair, or a series of estimates reflecting variation along the genome. Given a quantitative definition of RI (2.), how do we best measure it from empirical data, and how do existing approaches perform?

In this article, we will answer three main questions. First, What is RI? We will suggest a general definition of RI based on patterns of gene flow and explain its relationship with the organismal focus. Second, How can we define RI in precise quantitative terms? As RI is used as a quantitative concept (see above), this is crucial. Because the exact definition must depend on the scenario, we provide multiple quantitative definitions of RI, first in simple scenarios (two‐deme and continuous space), and then in examples of more complex scenarios that are more common in nature. Importantly, in our definitions we focus on the quantity that we think is most relevant in the context of speciation research a priori – not on the quantity that is most easily measurable empirically: A clear definition must be in place *before* aiming to measure RI empirically. Third, we therefore ask, How can we measure RI in practice? We discuss how to estimate RI from empirical data, asking whether, and under which circumstances, existing measures of RI reflect our quantitative definitions.

## 2 TOWARDS A GENERAL DEFINITION OF RI

**FIGURE 2:**
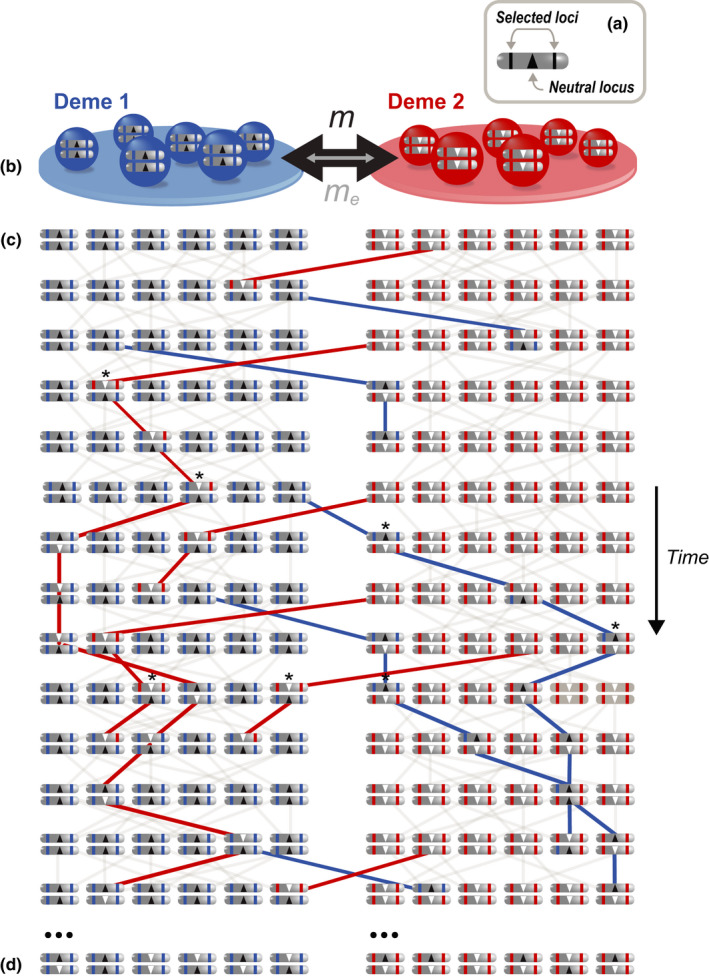
A pedigree depicting the movement of neutral alleles between populations and genetic backgrounds in the presence of a barrier. (a and b) Diploid individuals carry one chromosome with a single neutral marker flanked by two loci divergently selected between two demes. (c) At time 0 (top row), the two demes are fixed for alternative alleles at the neutral and selected loci. The blue and red selected alleles maximize fitness in demes 1 and 2, respectively, but severely reduce fitness in the other deme. Panel (c) depicts the pedigree for the 2 demes across multiple generations. Lines connect parents (row *i*) and their offspring (row *i* + 1). Coloured lines trace the passage of immigrant haplotypes through the pedigree. Because divergent selection is strong, selected immigrant alleles can only persist for a short time in the foreign deme. Because they are associated with selected sites, the movement of neutral alleles between demes is also restricted, but they can persist when they recombine onto the local genetic background. In this example, escaping their association with selected alleles requires two recombination events (asterisks mark individuals in which these recombination events occur). (d) Over time, the allele frequency difference at neutral loci will reduce, but this takes much longer than expected in the absence of a barrier

After some consideration and debate, we have settled on a general definition of RI not all that different to one posed by Dobzhansky (1937) or other gene flow‐based definitions (Gavrilets, 2004; Stankowski & Ravinet, 2021a) (For the definition of RI and other terms also see the Glossary, Table 1; Table S1). We chose to define RI in terms of gene flow, because the level of gene exchange is what ultimately determines the extent to which populations can evolve independently. We propose that RI is a quantitative measure of the effect of genetic differences on gene flow. RI compares the flow of neutral alleles from one population to another population, given a set of genetic differences that reduce gene flow, with the flow expected in the absence of any such differences (Figure 1B). The exact definition depends on the spatial context. By ‘population’, we simply mean a set of individuals. This could be defined by spatial position, but this is not necessarily so (see below). By ‘genetic differences that reduce gene flow’, we mean any genetic differences contributing to traits (at the organismal level) that restrict gene flow between groups of individuals, including both intrinsic incompatibilities and adaptations to local environments. These loci are often under divergent selection (see example in Figure 2), but can also include loci contributing to assortative mating or habitat choice.

**TABLE 1:** Glossary of key terms

Barrier to gene flow	A physical or genetic obstacle to gene flow.
Barrier loci	Loci that cause a barrier to gene flow.
Effective migration rate (*m_e_*)	The migration rate in the absence of a barrier that would have an effect equivalent to the actual migration rate in the presence a genetic barrier.
Geographic isolation	A reduction in migration between populations due to geographic barriers (e.g. mountains or rivers) or geographic distance.
Genome‐wide RI	The RI experienced by a neutral locus that is unlinked to any selected loci.
Local RI	The RI experienced by a neutral locus due to selection acting on both linked and unlinked selected loci. Local RI will inevitably vary from locus to locus, depending on the proximity to barrier loci.
Migration rate (*m*)	The fraction of individuals that derive from elsewhere in the previous generation.
Population	A group of individuals that are of some interest.
Reproductive isolation (RI)	RI is a quantitative measure of the effect of genetic differences on gene flow. RI compares the flow of neutral alleles from one population to another population, given a set of genetic differences that reduce gene flow, with the flow expected without any such differences. The exact definition depends on the spatial context, among other things.

Importantly, RI is defined only for neutral loci, not for the barrier loci (i.e. the loci contributing directly to the genetic differences that reduce gene flow, such as loci under divergent selection) themselves. Neutral loci can, however, be perfectly linked to loci contributing to barriers. We limit the definition of RI to neutral loci because we wish to separate selection on specific alleles from its effect on gene flow at linked and unlinked neutral loci.

While we define RI explicitly in terms of gene flow, it is directly connected to the ‘organismal focus’, as genetically based barriers are barriers to the production or fitness of hybrids. Genetically based barriers include, for example, intrinsic incompatibilities. If two populations contain incompatible alleles, fewer hybrids will be produced and thus the flow of neutral alleles between these populations will be reduced. Genetically based barriers also include those leading to assortative mating or habitat choice, if these barriers have a genetic basis and lead to a reduced probability of alleles moving between populations or areas. Importantly, genetically based barriers also include environment‐dependent barriers, i.e. loci contributing to local adaptation in a heterogeneous environment. For example, if large individuals are favoured by selection in one population and small individuals are favoured in another population, selection against migrants and hybrid individuals reduces the effective gene flow, generating RI.

In accordance with our definition, geographic isolation does not contribute to RI if it is not driven by genetic differences between populations. For example, geographical distance or a physical barrier (e.g. a mountain range or a river) reduces gene flow but is entirely environmental and not caused by genetic differences between populations, and thus does not contribute to RI. However, other geographical barriers *are* caused by genetic differences, for example spatial separation due to different habitat preferences (ecogeographic isolation, Box 2). In addition, physical barriers and genetic barriers can interact to modulate RI. We address both issues below.

BOX 2 **Ecogeographic isolation**

Sobel and Chen (2014) propose that when the differences between the ranges of two taxa are determined by genetic differences between them, the fraction which does not overlap should be included as a component of isolation. Here, we argue that the effect of range overlap on gene flow, and hence, on reproductive isolation, depends on both the geographic context, and on the nature of the genetic differentiation. Simple measures of range overlap are only informative under quite restrictive assumptions.

If the distinct populations have sufficiently different niches, and interbreed sufficiently rarely, then they may coexist in sympatry over some fraction of their ranges. If their respective habitats each form a mosaic, with some degree of overlap, and if there is substantial gene flow amongst patches of each of the two populations (Nm>>1), then we can treat them as two well‐mixed populations (Figure B2.1A). The effective migration rate will then be the fraction of range overlap, multiplied by the effective migration rate when they are in sympatry. In this situation, then, the fraction of non‐overlap is a sensible component of reproductive isolation, as proposed by Sobel and Chen (2014).

Next, suppose that the two populations have contiguous ranges, which overlap in an intermediate region along a one‐dimensional continuum (see Figure B2.1B for the corresponding 2‐dimensional scenario). This region can be treated as a localized barrier to gene flow, which causes a step in the frequency of divergent neutral alleles. If the density of one population is $n_{1}\left[x\right]$, which declines from $n_{1}^{*}$ on the left to zero on the right, and the other density is $n_{2}\left[x\right]$, which increases from zero on the left to $n_{2}^{*}$ on the right, then one can show that the barrier strength is $B=\left(\sigma^{2}n_{1}^{*}n_{2}^{*}\right)/m\int 2n_{1}n_{2}dx$. Here, *m* is the proportion of each population that is exchanged between the populations per generation (Figure B2.1; derivation in Appendix S2, Ecogeographic isolation). If there are additional incompatibilities, then *m* should be replaced by the appropriate effective rate. Thus, across a one‐dimensional habitat, the barrier strength *B* is the appropriate measure of reproductive isolation; if the integral in the denominator is taken as a measure of the distance over which the taxa overlap, then the barrier is inversely proportional to the fraction of range overlap.

It may be, however, that the taxa are separated by a narrow hybrid zone (Figure B2.1C), or by a broad region of intergradation (Figure B2.1D), with multiple overlapping clines. In such a case, the barrier may be calculated as described in the previous sections, and may be much weaker than suggested by the net divergence. With more than a few loci, the ranges of the parental genotypes will not overlap at all, even if the clines coincide (Figure B2.1C). Yet, as we have seen, a narrow hybrid zone may pose a negligible barrier to gene flow, across most of the genome; if the clines are scattered, the barrier will be still weaker (Figure B2.1D). In such cases, the fraction of overlap (however defined) is not an appropriate measure of isolation.

**Figure:**
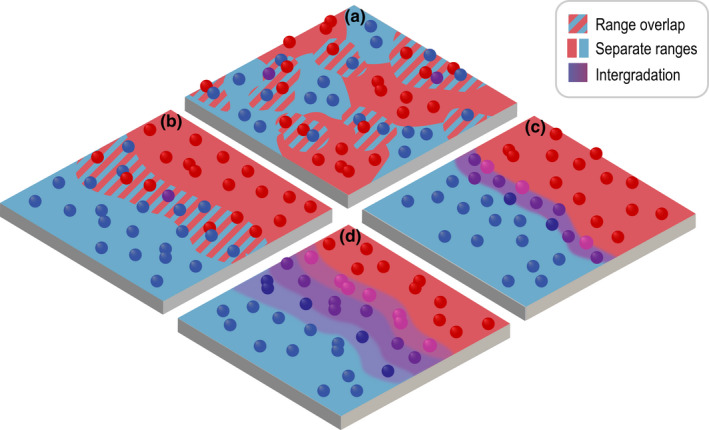
**FIGURE B2.1** Different patterns of range overlap, in a two‐dimensional habitat (Note that equations in the text are for a 1‐dimensional setting for simplicity). (a) Two populations (red, blue) are distributed in a mosaic, and remain distinct where they overlap. (b) Two contiguous ranges overlap in an intermediate region, again remaining distinct in an intermediate region of sympatry. (c) Two populations are separated by a narrow hybrid zone, in which clines for multiple genetic differences coincide. (d) Clines are scattered, so that there is a broader region of intergradation

In any given situation, three things are needed to formulate a concrete definition of RI: (i) the two populations, (ii) the timescale over which the allele movement is to be considered, and (iii) the genomic position of the focal neutral allele.

How to define the two populations is not necessarily obvious. First, groups can be defined spatially. Below we describe RI in a simple system of two demes connected by gene flow. In that case, it is obvious that the two demes correspond to the two populations. In arbitrarily complex spatial settings, it may be necessary to specify two areas between which we want to determine RI; the estimate of RI may differ substantially depending on which areas we choose. Second, populations could be defined by traits. For example, if two hybridizing taxa occur in sympatry, a trait that typically diagnoses them (e.g. plumage colour) could be used. Third, populations could be genetic clusters. For example, if two cryptic species occur in sympatry, genetic markers could be used to identify two genetic groups (e.g. using Principal Component Analysis or STRUCTURE; Pritchard et al., 2000). It is important to note that any grouping will be somewhat arbitrary: the fact that there is gene flow between the two ‘populations’ means that there are not actually two clearly separated groups. Reflecting the same issue, different groupings would typically lead to different results. For example, as alleles underlying traits can introgress, and as an individual in a deme might be a migrant, space‐ or trait‐based clusters may not be identical to genetic clusters. Many environments are complex, local adaptation to various environmental factors occurs, and different environmental transitions or gradients do not necessarily coincide in space. In such cases, there are multiple possible populations between which we could measure RI, and a single value is certainly not sufficient to generally describe patterns of RI.

Similarly, the temporal scale might not be straightforward to define. In scenarios where selected loci are at migration‐selection equilibrium (see examples below), the rates of gene flow stay constant over time, and no specific timescale needs to be defined. However, when selected loci are not at equilibrium, e.g. after a secondary contact between divergent populations, allele frequencies at selected loci change over time (e.g. while incompatibilities are purged or uniformly favoured alleles introgress) and thus the rate at which neutral alleles are exchanged may change as well.

Finally, it is crucial to define the focal genomic region for which the reduction in gene flow is determined. In the literature, RI is mostly discussed as a genome‐wide concept, with a single measure for a pair of populations or a hybrid zone (e.g. Lowry et al., 2008; Rabosky, 2016; Schluter, 2009). However, RI is also sometimes described as related to the effective migration rate, *m*_e_, which varies along the genome, suggesting that RI varies along the genome as well (Barton & Bengtsson, 1986). RI as a single genome‐wide concept must reflect the general barrier to gene flow experienced by a locus without any specific features – that is, a neutral locus unlinked to any selected loci. In contrast, RI along the genome reflects how effective gene flow varies between neutral loci along the genome depending on their association with particular loci contributing to reproductive barriers. We refer to these different concepts of RI as ‘genome‐wide RI’ and ‘local RI’, respectively.

In the following, we first provide simple examples where an exact quantitative definition of RI can be given. Again, our goal in this part is defining what quantities we are interested in when trying to understand the speciation process, independent of whether and how they can be measured in practice. How RI can be measured in reality is a separate question and will be discussed in a later section (Estimating RI from empirical data).

## 3 EXAMPLE SCENARIO 1: GENE FLOW INTO A SINGLE DEME

### 3.1 Assumptions

The simplest case is a situation with two populations (demes) with unidirectional gene flow from the source population into the focal recipient population. RI is generated by a set of selected loci that show fixed allelic differences between the source and the recipient population. We assume that the rate of gene flow is low, such that F_2_ hybrids are rarely generated and can be neglected, that our assumption of fixed differences at selected loci is a good approximation, and that the processes under unidirectional gene flow (assumed for simplicity) approximate those under bidirectional gene flow.

### 3.2 Quantitative definition of RI under this model

Gene flow between demes is described by a migration rate, *m*, which reflects the proportion of migrants in the focal deme after migration. This migration rate describes migration in the absence of reproductive barriers between demes. However, if reproductive barriers do exist, the actual rate at which neutral alleles from the source reach the recipient population is reduced, e.g. because they are associated with alleles that are selected against in the recipient population (Figure 1). They will in that case be more likely to be removed from the recipient population than in the absence of barriers, which is mathematically equivalent to a lower *effective* migration rate, *m*_e_ (Barton & Bengtsson, 1986; Bengtsson, 1985). Analogous to the baseline migration rate, *m*_e_ is simply the allele frequency change due to the arrival of migrants in a generation (δp), relative to the allele frequency difference between demes (Δp), $m_{e}=\delta p/\Delta p$.

Bengtsson (1985) describes the effective migration rate as ‘that rate of migration which would have the same evolutionary effect in a population with no genetic barrier as the actual migration rate (*m*) now has in the population with a barrier’. The effective migration rate is more strongly reduced for neutral loci closely linked to selected loci and increases with increasing distance from such loci.

The more *m*_e_ is reduced compared to *m*, the stronger is RI. In the described scenario RI (in this case labelled ${\text{RI}}_{2d}$ for ‘2 deme’) can therefore be defined through the ratio between *m*_e_ and *m*:

(1) $${\text{RI}}_{2d}=1‐\frac{m_{e}}{m}$$

If a locus is not affected by a barrier to gene flow, $m_{e}=m$, and ${\text{RI}}_{2d}=0$. If gene flow at a locus is completely prevented, $m_{e}=0$ and ${\text{RI}}_{2d}=1$.

In the limit of low migration rates, and when the population is at a genetic and demographic equilibrium, this quantity depends only on the source and recipient genotypes. In the following, we describe how both genome‐wide and local RI can be calculated under this model.

### 3.3 Genome‐wide RI: RI due to unlinked loci

We first show how to calculate RI if all selected loci are unlinked. Genome‐wide RI is defined by gene flow at a focal neutral locus that is not linked to any selected loci. Migrants enter the recipient population at a rate *m*, and alleles at the focal neutral locus enter the recipient population at the same rate. To find *m*_e_, we need the probability that an allele coming from the source population recombines onto the recipient genetic background. As *m*_e_ reflects an average over time rather than a snapshot, we need to consider this probability not just for the first generation after a neutral allele enters the recipient population, but for all following generations as well. Thus, *m*_e_/m is the probability that an allele in a newly arrived migrant will ultimately recombine onto the recipient genetic background. (This is essentially the reproductive value (Fisher, 1930; Grafen, 2006) of a fresh migrant, relative to that of native individuals.)

When first entering the recipient population, the allele experiences selection because it is located in an individual with a complete source population genome, thus containing all divergent alleles selected against in the recipient population. This individual will have a fitness ${\bar{W}}_{0}<1$ (relative to pure individuals of the recipient population, which will have a fitness of 1). ${\bar{W}}_{0}$ includes reductions in fitness due to all possible genetic differences, including reductions in viability, mating success and fecundity. In the following generation, the allele will be located in F_1_ hybrids between source and recipient genomes. These F_1_ hybrids still carry alleles from the source population, but the number of such alleles is halved compared to first‐generation migrants, so that selection is weaker; in addition, there might be heterosis. Importantly, because we assume that gene flow is low (see above), the focal allele (if still present) will be located in backcrosses with native individuals in all following generations, with fewer and fewer source alleles. Thus, the negative selection experienced by the focal neutral allele weakens over time as it becomes progressively decoupled from other source alleles. Because we are focussing on an unlinked neutral allele, all that matters in this scenario is the average fitness of backcrosses over successive generations.

As *m*_e_ describes the combined effect of the migration rate *m* and the probability to persist in the recipient population despite selection, it is simply $m_{e}=m{\bar{W}}_{0}{\bar{W}}_{1}{\bar{W}}_{2}\ldots$ (where ${\bar{W}}_{0}$ is the fitness of migrants, ${\bar{W}}_{1}$ the fitness of F_1_ hybrids, ${\bar{W}}_{2}$ the fitness of first‐generation backcrosses of F_1_ hybrids with native individuals, etc.).

RI is then

(2) $${\text{RI}}_{2d}=1‐\frac{m_{e}}{m}=1‐{\bar{W}}_{0}{\bar{W}}_{1}{\bar{W}}_{2}\ldots$$

(Bengtsson, 1974, chap. 3; 1985). This equation simply shows that ${\text{RI}}_{2d}$ increases with decreasing hybrid fitness.

For all generations, it is important to note that the relevant fitnesses are those of the actual migrant or hybrid genotypes in the recipient population, and that hybrid genotypes are modified by selection (through progressive purging of locally deleterious alleles). Thus, these ${\bar{W}}_{i}$ are not necessarily identical to hybrid fitnesses determined e.g. in lab backcrosses.

In Appendix S2 (Flow into a single deme, Unlinked loci), we show that RI in this scenario is primarily determined by the first few generations, and can roughly be simplified to $1‐{\bar{W}}_{0}{\bar{W}}_{1}{\bar{W}}_{2}^{2}$: The influx of genes is reduced by the same factor in the first backcross generation as in all subsequent generations.

The key message of this section is that we can calculate the effective migration rate, and thus a measure of RI, from the mean fitnesses of successive backcross generations. We have described a two‐deme situation, but a similar approach will work for two taxa in complete sympatry if the level of gene flow between them is low.

### 3.4 Local RI: RI along the genome

To understand local RI as it varies along the genome, we need to consider linkage. Compared to unlinked neutral loci (previous section), neutral loci linked to selected loci are more strongly affected by selection; to persist in the recipient population they must recombine onto the recipient genetic background before being eliminated by selection (Figure 2). Thus, the key parameter is the strength of selection, relative to recombination.

The simplest case is a focal neutral locus linked to a single selected locus with selection coefficient *s*, separated by a recombination distance *r*. Barton and Bengtsson (1986) show that in this case

(3) $${\text{RI}}_{2d}=1‐\frac{r}{s+r}$$

**FIGURE 3:**
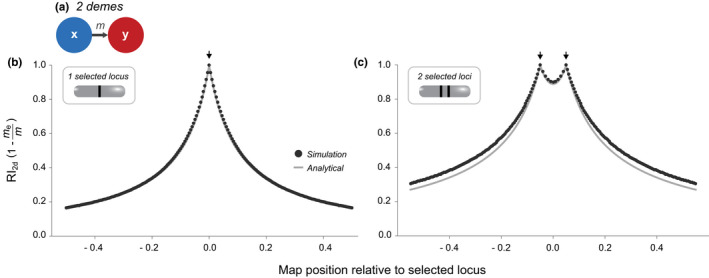
Reproductive isolation in a two‐deme scenario. (a) Two panmictic demes connected by unidirectional gene flow; the two locations between which RI is measured simply correspond to the two demes. (b and c) RI along the genome under this model, with a single locus under selection at position 0 (b) or two loci under selection at positions −0.05 and 0.05 (c). The *x*‐axis gives the recombination rate of the neutral locus relative to position 0.0 (−*r* for loci to the left and +*r* for loci to the right). The black curve corresponds to deterministic simulations (details in Appendix S1); the grey curve corresponds to Equation (4). Note that RI is not defined for the selected loci themselves; the points corresponding to the positions of the selected loci represent neutral loci perfectly linked to selected loci. *s *= 0.1 for all selected loci

This equation demonstrates that a neutral locus tightly linked to the selected locus (r≪s) experiences a strong barrier, and ${\text{RI}}_{2d}$ approaches 1 (under the assumptions of our model, including fixed or nearly fixed differences at the selected locus). Gene flow is reduced substantially within a region of size r≈s (Figure 3), and ${\text{RI}}_{2d}$ decreases with increasing distance from the selected locus. However, even unlinked loci (i.e. *r *= 1/2) induce ${\text{RI}}_{2d}∼2s$, consistent with the previous section, showing that even without linkage to selected loci, considerable RI can be produced.

Next, suppose that there is one selected locus on either side of the focal neutral locus, with selection and recombination rates on the left (*s*_1_, *r*_1_) and on the right (*s*_2_, *r*_2_), and the selective effect of the two alleles together (*s*_1,2_). The effective migration rate is now the product of the effects of each locus, multiplied by a term which equals 1 if there is no epistasis (i.e. $s_{1,2}=s_{1}+s_{2}$):

(4) $${\text{RI}}_{2d}=1‐\frac{r_{1}}{r_{1}+s_{1}}\frac{r_{2}}{r_{2}+s_{2}}\frac{r_{1}+r_{2}+s_{1}+s_{2}}{r_{1}+r_{2}+s_{1,2}}$$

(from equations (A6), (A7) of Barton & Bengtsson, 1986). This equation shows that, assuming a constant distance between the two selected loci, a neutral locus equidistant from both selected loci experiences the lowest levels of RI, while a neutral locus tightly linked to one of the selected loci can experience a much stronger barrier, even though it is further away from the second selected locus (Figure 3).

Finally, there might be multiple selected loci on either side of the focal neutral locus. For a given set of selection coefficients and recombination rates, RI can readily be calculated, but there is no simple general equation describing RI when the number of selected loci is large. Also in this case, the ratio of selection to recombination is a crucial parameter. RI decreases the further the selected loci are apart and (as in the previous example) the further away the neutral locus is from the nearest selected locus (see Appendix S2, Flow into a single deme, Linked loci, for further details).

In this section, we considered the effects of divergent selection on tightly linked loci; previously, we considered unlinked loci. In reality, neutral loci will often be affected by both linked and unlinked selected loci. Their relative contributions to RI for an average neutral locus depend on the length of the genetic map, and on the number of selected loci. Consider the simplest case, where selection is spread uniformly over a map of length *R*, with total multiplicative selection S=θR. In this case, with relatively strong selection (i.e. large *θ*; S>>R), unlinked loci make a larger contribution to the RI the neutral locus experiences; linked loci would dominate only for small or moderate *θ* (≤1, say), and for extremely large numbers of loci ($\log\left[n\right]>R$) (see Appendix S2, Relative contributions of unlinked vs. linked loci). However, these two components of reproductive isolation act over different timescales: selection over the whole genome (which is mostly unlinked) acts to quickly reduce the contribution of migrants, by severely reducing the fitness of early generation backcrosses (see above). Selection at tightly linked loci then acts over a much longer timescale, because it takes longer for recombination to break up the association between the selected and the neutral loci. Even if linked selection is responsible for only a small fraction of reproductive isolation, it may still cause ‘islands of divergence’ around the selected loci.

## 4 EXAMPLE SCENARIO 2: HYBRID ZONES

### 4.1 Assumptions

Where divergent populations meet in continuous space, rather than in a two‐deme situation (previous section), they may be separated by hybrid zones, i.e. they locally interbreed and form hybrids, but remain distinct away from the hybrid zone centre (Barton & Hewitt, 1985; Bazykin, 1969; Stankowski et al., 2021). We here assume a hybrid zone in a single dimension. The reduction in gene flow across the zone can be caused by intrinsic (e.g. Dobzhansky‐Muller incompatibilities) as well as extrinsic (e.g. ecological) components, but importantly, we assume that all genetic barriers to gene flow more or less coincide in space. We also again assume that an equilibrium between migration and selection has been reached for the selected (but not necessarily the neutral) loci.

Under these conditions, spatial clines form at selected and neutral loci. A cline is a gradual change in allele frequency that changes sharply in the centre of the zone and decays more gradually towards both sides (see Figure 5 for examples from simulations). The steepest clines form for selected and closely linked loci.

### 4.2 Quantitative definition of RI under this model

If the population is distributed along a spatial continuum, we cannot unambiguously define two populations between which to determine RI. We could arbitrarily define two areas and use the effective migration rate as above, but the result would then depend strongly on how the two populations are delimited. It is thus more natural to view gene flow as a diffusion across continuous space (Fisher, 1937; Haldane, 1948; Wright, 1943) rather than as a rate between two arbitrarily defined demes, and to measure reproductive isolation through its effect on the rate of diffusion across the hybrid zone itself.

In continuous space, the rate of diffusion of alleles depends only on the variance of the spatial distance between parent and offspring (Nagylaki, 1976). To fit the general definition given in the Introduction, RI must describe how this diffusion process is impeded by genetic differences between the two sides of the hybrid zone. In a simple model of diffusion, the only factor that affects gene flow between two points is the spatial distance between these points. It is thus most natural to represent RI with the unit of a distance, where a greater distance implies a greater obstacle to gene flow.

For that, one can calculate the spatial distance that would be needed to generate the same allele frequency change if there was no barrier to gene flow. The allele frequency change without a barrier is represented by the gradient of allele frequency (or slope of allele frequency change), *p*′, near the barrier (Figure 5C). If there was no barrier, allele frequency would continue to change as a straight line with that slope. However, the local barrier ‐ whether it be physical or genetic ‐ causes an abrupt step in allele frequency, Δp (Figure 5C). Thus, the strength of the barrier to gene flow can be defined as $B=\Delta p/p^{\prime }$, i.e. the distance that would be required to generate the same allele frequency change as the step (Figure 5C). Crucially, this ratio settles to a constant value within a short amount of time (Nagylaki, 1976) (e.g. after the emergence of a new selected mutation or secondary contact between diverging populations).

This distance, *B* (for barrier), for any neutral locus must depend on how closely that neutral locus is linked to selected loci and on the strength of selection. Accordingly, Barton (1986) showed that *B* is approximately

(5) $$B=\int \left({\left(\frac{\bar{W}(x)}{{\bar{W}}_{0}}\right)}^{‐\frac{1}{r}}‐1\right)dx$$

where *r* is the harmonic mean recombination rate between the focal neutral locus and all the selected loci, and $\bar{W}(x)$ is the mean fitness at position *x* in the hybrid zone, relative to the mean fitness outside, ${\bar{W}}_{0}$ (note that this is a different ${\bar{W}}_{0}$ from the one defined in the two‐deme context above). The equation for *B* is an integral (a sum) over all positions *x*, so that *B* reflects the barrier across the whole hybrid zone. This equation is an approximation valid in the limit where selection is weak relative to recombination, but remains accurate for moderately strong barriers (provided that the distribution of hybrid index is unimodal; Barton & Shpak, 2000; Kruuk et al., 1999).

**FIGURE 4:**
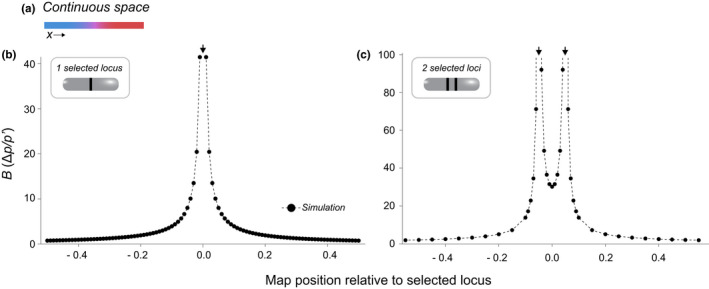
Reproductive isolation in a continuous space scenario (across a simple hybrid zone). (a) Two divergent populations meet in a hybrid zone in continuous space. (b and c) The barrier *B* along the genome under this model, with a single locus under selection at position 0 (b) or two loci under selection at positions −0.05 and 0.05 (arrows) (c). The *x*‐axis gives the recombination rate of the neutral locus relative to position 0.0 (−*r* for loci to the left and +*r* for loci to the right). Points show results from deterministic simulations, and the line connects these points (details see Appendix S1). *s* = 0.1 for all selected loci

As it depends on recombination rate, this equation can be applied for linked as well as unlinked neutral loci and can thus be used to calculate genome‐wide as well as local RI. Figure 4 shows an example of how *B* changes along the genome. *B* increases with decreasing hybrid fitness and with proximity of the focal neutral locus to selected loci.

*B* describes the impediment to movement of neutral alleles from one side of the hybrid zone to the other, given divergent selection at some loci, as a spatial distance. The comparison to the movement without any such barriers is implicit, as in that case the corresponding spatial distance is just 0. *B* therefore fits our general definition of RI but differs from the definition of RI given for the two‐deme model in that it can exceed 1 and has the unit of a spatial distance.

### 4.3 RI between two demes versus across a hybrid zone

What is the relation between measures of RI in a two‐deme vs. a hybrid zone situation, i.e. the relation between ${\text{RI}}_{2d}=1‐\left(m_{e}/m\right)$ and *B*?

We can determine *m*_e_ and relate it to *B* in a hybrid zone if we consider a zone that is limited in space (i.e. individuals to the left and right of the hybrid zone centre occupy a finite spatial range) and established a long time ago. Under these conditions, allele frequencies for neutral loci on each side of the central step are almost constant due to long‐term mixing. The left and right side thus naturally form two areas similar to two demes, which are separated by a barrier to gene flow, and between which an effective migration rate can be determined (see Appendix S2, Relation between *m*_e_ and *B* in one dimension):

(6) $$m_{e}\approx \frac{\sigma^{2}}{2BX},$$

where *X* is the length of the habitat on the focal side of the zone. Thus, under these conditions, *m*_e_ simply increases with the inverse of the barrier strength.

However, to determine RI analogous to a two‐deme model, we not only require *m*_e_ but also *m* for the hybrid zone. How to define *m* for a hybrid zone is less clear: While two homogeneous areas, similar to two demes, can naturally emerge for neutral loci affected by a barrier to gene flow (see above), this is not the case for neutral loci not affected by a barrier to gene flow (which would be required to determine *m*). Thus, it is unclear between which two areas *m* should be defined, and there is no natural direct equivalent to ${\text{RI}}_{2d}=1‐\left(m_{e}/m\right)$ for a hybrid zone (This issue is elaborated in Appendix S2, Relation between *m* and *B* in one dimension).

Nevertheless, empirical studies frequently apply two‐deme models and assumptions to systems that actually show divergence across a continuous hybrid zone (e.g. studies calculating *F*_ST_ along the genome for two samples from hybridizing populations). Samples are often taken at a somewhat arbitrary distance from the hybrid zone centre. A main reason for doing this is that hybrid zone analysis usually requires much larger sample sizes, and spatially extensive sampling. It is important to notice that in this case, estimates of *m*_e_ or ${\text{RI}}_{2d}$ reflect the reduction in effective gene flow between the two sampled areas, which is not necessarily the same as the reduction experienced directly in the hybrid zone. If the samples used are distant from the hybrid zone, it is possible that additional barriers to gene flow located in space between the hybrid zone centre and the samples contribute to the measure of *m*_e_ or ${\text{RI}}_{2d}$. ${\text{RI}}_{2d}$ thus summarizes the effects of all barriers between the two samples, and is not equivalent to *B* for the hybrid zone.

## 5 OTHER SCENARIOS

### 5.1 Simulations can be used to determine RI in a multitude of scenarios

**FIGURE 5:**
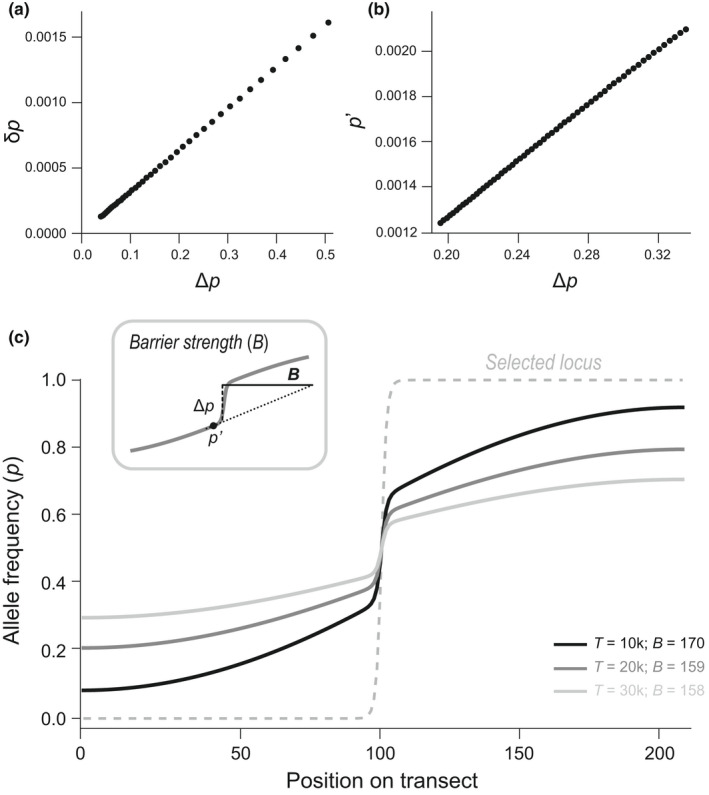
Estimating RI from simulations. (a) Discrete demes: In contrast to most empirical situations, in simulations it is possible to observe not only the allele frequency differences between two demes in a given generation (Δp) but also the allele frequency change over a generation (δp). As the ratio between the two gives *m*_e_, the slope of δp against Δp provides *m*_e_. The different values in the plot can come either from following a single neutral locus over time or from looking at multiple loci with different allele frequencies across a single generation; but note that the linear relationship between δp and Δp from different time points will only appear when the populations are at equilibrium for the selected loci; out of equilibrium, the time point to determine RI must be specified and all values must be sampled from that same time point. In either case, RI can simply be calculated from *m*_e_ and the known *m*. (b) Hybrid zone: The barrier *B* for a neutral locus can be measured as the ratio between the gradient (*p*′) and the central step (Δp) (see panel (c)), which also stays constant over time. Again, the different values in the plot can come either from following a single neutral locus over time (as in panel (c)) or from looking at multiple loci with different allele frequencies in a single generation. (c) Hybrid zone: RI can be measured from cline patterns. At the selected locus (grey dashed line), a steep spatial cline has reached equilibrium. At linked neutral loci, the barrier to gene flow generates weaker clines. We show a single linked neutral locus at three time points (different shades of grey). After an initial short period of stabilization (not shown), even though neutral allele frequencies are still changing, the barrier *B* measured for the linked neutral locus is approximately stable over time, reflecting a stable rate of flow across the zone. Simulations with nearest neighbour migration with m=0.5, s=0.2 for the selected locus and r=0.01 for the neutral locus

Above we have discussed quantitative definitions of RI under two relatively simple scenarios, making simplifying assumptions. These assumptions may be violated in many empirical settings. The exact effects of these violations on RI can in some cases be explored analytically (e.g. effects of >2 demes; see below). In other cases, this might not be currently easy (e.g. a two‐deme scenario with high migration; see below). However, importantly, RI can be evaluated in any scenario that can be simulated. For discrete demes, both the change in allele frequency at a focal neutral locus in a focal deme (δp) and the allele frequency difference between demes (Δp) can be recorded in simulations and provide $m_{e}=\delta p/\Delta p$ (Figure 5A). As the migration rate *m* is set for the simulation, RI can be calculated. In continuous space, for a hybrid zone situation, *B* can be measured as $B=\Delta p/p^{\prime }$ (Figure 5B,C). In more complex settings in continuous space, one can arbitrarily define two areas between which RI can again be measured using δp and Δp.

We cannot cover all deviations from the simple two‐deme and hybrid zone models in this paper. However, in the following, we discuss the effects of deviations that may be particularly common in nature.

### 5.2 Range overlap

The two focal populations may occupy broadly overlapping ranges, living in (partial) sympatry; this is distinguished from overlap in a narrow hybrid zone that is maintained by a balance between dispersal and selection (see above). For this situation to be stable, there must be genetic differences that prevent the populations from mixing, and consequently, some degree of reproductive isolation. The effective migration rate and RI can be defined in the same way as for two demes in different locations. In Box 2, we consider this situation in more detail, and in particular, whether the fraction of range (non)overlap is an appropriate measure of RI.

### 5.3 Two‐deme models with higher gene flow

If levels of gene flow between two demes are high (as opposed to our assumptions above), F_2_ and complex hybrids may be common, and the outcome is unpredictable. We cannot simply focus on the fitness of successive backcrosses, but need to consider the fitnesses of all possible hybrid genotypes and their respective frequencies, making the calculation more complicated. There is also a more fundamental problem: with very high levels of gene flow, what are the two populations between which we want to measure RI? If most individuals cannot clearly be assigned to a population, a measure based on two distinct groups may not be appropriate. This issue poses a fundamental challenge for any concept of RI, as it implies that RI is often hard to define at the early stage of the divergence process.

### 5.4 Gene flow between two demes within a larger set of demes

If we define a set of demes each with its own set of selection coefficients and connected by a set of migration rates, we can still define the effective migration rate. For that, the analytical approach proposed by Barton and Bengtsson (1986) splits all possible individuals into a set of ‘pools’, where each deme – genetic background combination is a different pool (genetic background = genotype at selected loci). Then, one can generate a (potentially very large) matrix that describes the gene flow for each possible pair of such pools for a neutral locus; the matrix values are dependent on *m*, the selection coefficient on each genotype in each given deme, and the recombination rates. *m*_e_ between pairs of demes at equilibrium can then be calculated from this matrix.

### 5.5 Non‐equilibrium situations

We have assumed the selected loci to be at equilibrium (or fixed different) when defining RI in specific situations above. In that case, RI is stable over time, even though allele frequencies at neutral loci might change – their long‐term rates of gene flow are constant, and thus RI is constant as well.

If selected loci are not at equilibrium, RI will change over time. A simple example to see this is a secondary contact between two demes containing multiple intrinsic incompatibilities of the form AAbb in deme 1 and aaBB in deme 2, with A and B being incompatible but otherwise not selectively different from a and b. These incompatibilities can be resolved by fixing the ancestral alleles (a and b) in both populations. Thus, immediately upon secondary contact, there might be a strong barrier to gene flow, but over time this barrier will dissolve.

Note, again, that it is only relevant whether the loci contributing directly to the barrier have reached equilibrium. It is irrelevant whether neutral loci are at equilibrium, as *m*_e_ is independent of the frequency of the neutral alleles; for example, *m*_e_ for an unlinked neutral locus is the same independent of whether that locus has very recently obtained a new mutation in deme 1 or shows a large allele frequency difference between deme 1 and deme 2.

In non‐equilibrium cases, it is important to define the time point/time interval at which we want to determine RI. One can then (at least in principle) calculate or simulate the migration matrix as it changes through time, and thus also calculate how RI changes over time. The analytical approach proposed by Barton and Bengtsson (1986) described above can be used to calculate *m*_e_ between demes for any given time point. However, importantly, in a non‐equilibrium situation, the frequency of different genetic backgrounds (for selected loci) changes from generation to generation, and therefore this matrix changes over time as well, making the calculation more complicated.

### 5.6 Heterosis and adaptive introgression

Frequently, F_1_ hybrids are fitter than either parent. In the short term, such heterosis will increase the effective migration rate, by e.g. increasing ${\bar{W}}_{1}$ in Equation (2) (which still applies). However, if the heterosis is due to the presence of different deleterious recessives in the two populations, then it will be transient, since gene flow and selection will together tend to equalize allele frequencies. This is a special case of a more general phenomenon: when an allele that is favoured in both populations sweeps across, it will take with it a surrounding block of genome, thereby *increasing* neutral gene flow rather than reducing it, as discussed in most of this article. RI can in these cases be negative. If there is recurrent selection at the same locus, there may be a long‐term increase in gene flow, and a consistent reduction in RI. One example of this phenomenon may be the frequent observation of introgression of mitochondrial DNA in animals, which inflates the flow of all the alleles carried on these maternally inherited organelles (Sloan et al., 2017; Toews & Brelsford, 2012).

### 5.7 Effect of a physical barrier

As mentioned above, it is important to note that a physical barrier to gene flow (e.g. a river, wall or mountain, or a local reduction in population density due to e.g. less available habitat) does not directly contribute to RI, as it is not based on genetic differences. However, it does restrict gene flow. In a two‐deme setting, a physical barrier between demes just reduces *m*. In continuous space, the situation is more complicated, because the dispersal rate is only reduced in a specific area. In that case it is possible to calculate a $B_{\text{phys}}$ for a physical barrier analogous to that for a genetic barrier (Barton, 1986) (Appendix S2, Effect of a physical barrier).

**FIGURE 6:**
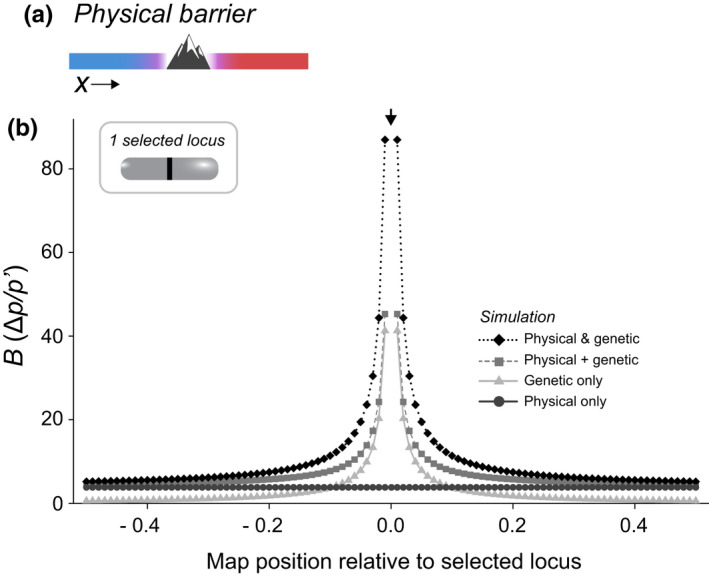
Reproductive isolation in continuous space, with a physical barrier. (a) Two divergent populations meet in a hybrid zone in continuous space; the genetic barrier coincides with a physical dispersal barrier. (b) The barrier *B* along the genome under this model, comparing different situations with and without physical and genetic barrier. Circles: Only a physical barrier, with no selection; Triangles: Only a genetic barrier; Diamonds: the genetic and the physical barrier acting together; Squares: For comparison, the (hypothetical) barrier that would appear if the genetic and the physical barrier just added up. This plot shows the synergy between the physical and genetic barrier. The *x*‐axis gives the recombination rate of the neutral locus relative to position 0.0 (−*r* for loci to the left and +*r* for loci to the right), which corresponds to the position of the selected locus with *s* = 0.1, if present. See Appendix S1 for details

Importantly, even though a physical barrier itself is not part of RI, co‐located physical and genetic barriers interact to together form a stronger barrier than expected from just adding up the two barriers (i.e. two values of *B*) (Figure 6).

## 6 Estimating RI from empirical data

Above, we have provided definitions of RI in different spatial settings. In the empirical literature, there are various approaches for estimating RI. They differ particularly in whether they focus on the genetic or organismal level, and on whether they generate a single estimate of RI (genome‐wide RI) vs. multiple estimates along the genome (local RI). In the following, we summarize existing approaches, discuss how they relate to the above definitions and to each other, and discuss under what conditions they measure RI, as we have defined it. We also highlight some promising approaches to estimating RI that may overcome some of the limitations of current methods.

As discussed above, in non‐equilibrium situations (for selected loci) the value of RI will depend on the timescale considered. Different measures of RI implicitly focus on different timescales, and will thus often provide different estimates. Organismal methods, based on immigrant fitness and the fitness of the first few hybrid generations, measure RI on very short timescales. Hybrid zone analysis applies in a limited spatial context, where selected and neutral loci typically equilibrate quickly, and thus reflects processes on a timescale of hundreds or thousands of generations. Methods based on sequence divergence can reflect processes over much longer timescales, of order the effective population size of the whole species.

### 6.1 Organismal measures of RI

Several measures of RI focus on the organismal level, without using genetic data. The most direct way to measure RI at this level is to estimate the fitness of migrants and hybrids experimentally or in the field. We discuss above how these fitnesses (${\bar{W}}_{0},{\bar{W}}_{1}\ldots$) relate to genome‐wide RI (Eq. 2, RI for an unlinked neutral locus in a two‐deme model under low gene flow). In a two‐deme situation, one would ideally determine the fitness of immigrants, F_1_ hybrids and backcrosses in each deme. It might be difficult to do this in laboratory experiments, as it will usually be hard or impossible to include all components of fitness. It might be more promising to use field data: using genetic markers, individuals in a deme can be assigned to the migrant, F_1_ or successive backcross generations (in practice, this works only for the first few generations, but the genome‐wide barrier depends mostly on these). This categorization can be performed in unmanipulated natural populations or after transplanting a set of ‘migrants’ into the recipient population and tracking their offspring (McBride & Singer, 2010; Schluter et al., 2021). The offspring numbers for the migrant, F_1_ and backcross individuals can be determined (e.g. by counting eggs or using pedigreed populations) and compared to that for the recipient population, thus producing a measure of relative fitness for each generation. If sample sizes are large, one could alternatively use the migrant and hybrid counts directly as a measure of fitness of the previous generation (However, this requires obtaining migrant numbers before selection). These approaches reflect RI over short timescales (only over the studied set of generations) and should provide more direct estimates than those from sequence divergence discussed below. Note that this approach does use genetic data to assign individuals to hybrid categories, but it uses a direct estimate of fitness (offspring number) to calculate RI. A shortcoming of this approach is that large numbers of genotyped individuals are necessary if hybridization is rare.

Previous work on organismal measures of RI has used definitions of RI deviating from what we propose here (Coyne & Orr, 2004; Ramsey et al., 2003; Sobel & Chen, 2014). In their classic meta‐analysis of *Drosophila* crosses, Coyne and Orr (1989) proposed simple measures of RI that they used to study the rate and order of appearance of prezygotic (mating isolation) and postzygotic barriers (F_1_ viability) in a comparative framework. For example, their measure of prezygotic isolation, based on lab mating experiments, was ${\text{RI}}_{\text{pre,CO}}=1‐\left(\text{frequency of heterospecific matings}/\text{frequency of homospecific matings}\right)$. Although Coyne and Orr (1989) did not discuss the relationship between these estimates of RI and their effect on gene flow, there is an underlying connection with *RI*_2D_. For example, if frequency of heterospecific matings/frequency of homospecific matings was measuring the relative fitness of immigrants, this would be equivalent to $1‐{\bar{W}}_{0}$ in Equation (2), above. However, in Equation (2), ${\bar{W}}_{0}$ reflects all of the barriers affecting the fitness of immigrants, not just mating isolation, so other barriers would also need to be quantified. Measuring and combining multiple barriers mathematically is straightforward in principle, but large numbers of experiments would be required to assess all potential barriers in practice. Even then, this measure does not include the fitnesses of the following hybrid generations (e.g. ${\bar{W}}_{1},{\bar{W}}_{2}\ldots$), which would be required for the measure of RI to reflect gene flow.

Sobel and Chen (2014) propose alternative measures of RI, which are similar to Coyne and Orr's (1989) measure. However, unlike Coyne and Orr (1989), who were more focussed on having a rough proxy for the relative strength of different barriers, Sobel and Chen (2014) indicate that the purpose of organismal measures of RI is to estimate how much gene flow is reduced by isolating barriers. They aim for a linear relationship between their measure and H/(C+H) (where *H* and *C* are the numbers of heterospecific and conspecific matings, respectively), which they consider the ‘probability of gene flow’. This probability is not the rate of gene flow into a deme but the probability of mating in a symmetrical contest, e.g. for the parental taxa when they fully coexist in sympatry. Sobel and Chen (2014) thus propose ${\text{RI}}_{\text{SC}}=\left(1‐\left(H/C\right)\right)/\left(1+\left(H/C\right)\right)$. They also suggest methods to calculate the total isolation by combining the effects of different barriers. Postzygotic isolation is included by setting *H* to the fitness of the offspring of heterospecific crosses and *C* to the fitness of the offspring of conspecific crosses. Similar to Coyne and Orr's (1989) measure, Sobel and Chen's (2014) measure of RI mostly focuses on mating between the parental taxa, while RI must depend also on the fitness of the following generations. For example, even if individuals of two taxa readily mate and produce fertile F_1_ offspring, many F_2_ or backcrosses might be sterile.

Another problem with the organismal measures of RI, like those proposed by Coyne and Orr (1989) and Sobel and Chen (2014), is that the estimates of RI are usually made out of the natural context of the study system. For example, even if a mating trial in the lab reveals that the probability of heterospecific matings in lab crosses is low, there may be so many mating opportunities in nature that almost all immigrants will eventually find a mate; then, at least female fitness might not be greatly reduced. The probability of mating in a single (lab) encounter thus may not reflect the long‐term reproductive success of an individual. It might be possible to obtain more realistic measures of the mating component of fitness by designing mating experiments closely mimicking field conditions.

A further issue is that individuals used in mating experiments and crosses are sometimes sampled far away from areas of hybridization and would never encounter each other in nature. This is most likely to happen in studies of taxa that have separate distribution ranges but form local unimodal hybrid zones. To ensure crossing the parental taxa rather than hybrids, researchers must sample away from the hybrid zone centre to avoid hybrids – but sampling *too* far from the zone centre is also problematic because barriers to gene flow can be resolved by displacing the clines of the contributing loci from the zone centre. This is expected if divergence involves Dobzhansky‐Muller incompatibilities (Orr, 1996). If a derived allele *A* is favoured at locus ***A*** in population 1, and a derived allele *B* is favoured at locus ***B*** in population 2, and alleles *A* and *B* are incompatible, then the ancestral alleles *a* and *b* will become common in the hybrid zone centre, alleviating incompatibility. There are several such examples in natural hybrid zones (e.g. Hatfield et al., 1992; Virdee & Hewitt, 1994). Clines at loci ***A*** and ***B*** will be pushed apart in space, so that there is no barrier to gene flow in nature because the incompatible alleles (*A* and *B*) do not meet. Measures of *m*_e_ based on genomic data (see below) will thus not indicate a genetic barrier to gene flow. However, when individuals sampled distant from the hybrid zone are crossed, they will contain incompatible alleles and give the misleading impression that there is a barrier to gene flow. While it is correct that an incompatibility exists, it is not relevant in the natural context and should thus not be incorporated in a measure of RI.

Another general limitation of organismal approaches is that they do not explicitly consider the genomic locations of selected and neutral loci; i.e. they cannot measure variation of RI along the genome, and instead (at best) give the expected reduction in gene flow for an unlinked neutral locus. Moreover, under high gene flow or in continuous space (for example, across hybrid zones) there are a multitude of fitness classes (each possible genotype at selected loci in each environment might have a different fitness), making the calculation of their effect on the net rate of gene flow very complicated; in such cases, the fitnesses of all hybrid genotypes cannot in practice be measured. We expect organismal methods to have the potential to provide meaningful RI estimates only in situations with populations connected by low levels of gene flow, either because of a large distance or physical barrier to gene flow, or because strong RI has already evolved and limits exchange.

In summary, as the organismal methods estimate migrant and hybrid fitnesses, genome‐wide RI can in principle be estimated directly from organismal measures by multiplying across independent fitness components and across generations (Eq. 2). This is only appropriate in cases where gene flow is low. It requires taking multiple generations and barriers into account; it is also important that lab mating probabilities are not equated to fitness components in the field unless the experiment is specifically designed towards this goal or an estimate of the fitness components can be calculated from the mating data (see Perini et al., 2020 for an example in this direction). In addition, hybrid and backcross fitness measured in the lab might differ from the field because deleterious alleles might be purged more effectively in the field (i.e. increasing ${\bar{W}}_{2}$, ${\bar{W}}_{3}$ … in the field); however, this error is small unless selection is very strong (see Section 3 and Appendix S2, Flow into a single deme, Unlinked loci).

A great advantage of organismal approaches over genetic ones is the opportunity to explore the actual barriers contributing to RI. For example, many studies have used the method outlined by Sobel and Chen (2014) to make the distinction between pre‐ and postzygotic or intrinsic and extrinsic barriers in individual cases of speciation (Briscoe Runquist et al., 2014; Lackey & Boughman, 2017; Mérot et al., 2017; Sobel & Streisfeld, 2015). These approaches complement genomic studies, which are often difficult to interpret, as approaches like *F*_ST_ scans can frequently generate signals of ‘barriers’ that in fact have nothing to do with RI.

### 6.2 Hybrid zone analysis (geographic cline analysis)

Unlike the above methods, which often drastically simplify or ignore the geographic setting, hybrid zone analysis uses spatial patterns of genetic variation to quantify the strength of barriers. In example scenario 2, we showed how a genetic barrier impedes the flow of neutral alleles through continuous space. This leads to the formation of a cline with a sharp central change of allele frequencies flanked by relatively shallow tails of introgression (examples in Figure 5). This pattern is seen in many natural hybrid zones (e.g. in *Bombina* toads (Szymura & Barton, 1986, 1991), pierid butterflies (Porter et al., 1997) and house mouse (Macholán et al., 2007)), and has been used to estimate the barrier strength, *B*, for neutral loci linked and unlinked to selected loci, as described above ($B=\Delta p/p^{\prime }$).

To calculate *B* in practice, one needs large sample sizes and dense spatial coverage to resolve the allele frequency gradients at the centre and edges of the zone. The size of the central step (Δp) and the slope of the flanking gradients (*p*′) must then be estimated. This is difficult to do directly, so a three‐part ‘stepped’ cline model (left gradient – step – right gradient) is usually fit to the data (Porter et al., 1997; Szymura & Barton, 1986). Because the left and right tails can be approximated by separate functions, *B* can be estimated separately for each side of the hybrid zone to quantify asymmetry in gene flow.

As cline shapes for selected loci quickly equilibrate when selection changes (on a timescale of ∼1/s), and on a local scale neutral alleles quickly respond to changes, *B* reflects gene flow typically over a timescale of hundreds to thousands of generations.

The interpretation of *B* may be difficult in practice because physical barriers have the same effect on patterns of gene flow as genetic ones (see above), so it is necessary to rule out or correct for their effect. Physical barriers are not always readily apparent (e.g. a mountain or river) and may include more subtle environmental features that make the landscape less permeable to dispersal or harder to inhabit (e.g. differences in soil chemistry or water salinity). Mapping of the population density and habitat variables may help reveal physical barriers (Barton & Hewitt, 1985; Hewitt, 1988), but density may also be reduced at a genetic barrier if selection against hybrids is strong (i.e. ‘hybrid sink’ effect; Barton, 1980). This makes it difficult to disentangle the effects of physical and genetic barriers, but conclusions may be strengthened by other lines of evidence, including inferences from multiple independent transects (e.g. Szymura & Barton, 1991) or direct measurements of dispersal (Barton & Gale, 1993).

Despite its strengths, geographic cline analysis also has some inherent limitations. The most obvious is that it cannot be applied to organisms that do not hybridize in nature, or those that form complex mosaic hybrid zones that do not show a smooth one‐dimensional cline (e.g. Bierne et al., 2003). Another practical challenge is that cline fitting is statistically delicate, and application to genome‐wide datasets is challenging. Moreover, cline shapes are not only affected by dispersal and selection, but also by genetic drift (Polechová & Barton, 2011); there is little understanding of how this affects estimates of *B* especially in small populations. Finally, *B*, calculated in the absence of other methods does not tell us anything about the types of barriers that cause RI and cannot easily be related to other measures of RI, like ${\text{RI}}_{2d}$.

Inferences about the strength of RI have also been made from the distributions of hybrid index (HI) scores from individuals sampled at the centre of hybrid zones (Gay et al., 2008; Irwin, 2020; Jiggins & Mallet, 2000). Because HI scores are calculated from numerous unlinked loci that diagnose different taxa, the distribution of scores in areas of overlap, which summarize multilocus patterns of LD, must reflect the historical local rate of production and fitness of hybrids. For example, complete isolation in sympatry will maintain maximum LD among loci, so that HI scores remain perfectly bimodal. In contrast, the absence of any barriers will cause LD to decay, resulting in a unimodal distribution. Partial isolation is expected to result in a distribution somewhere in between. However, hybrid zones are usually just classified by their modality, thus not producing a quantitative measure of RI, and the genome‐wide barrier to gene flow has to be very strong to maintain a bimodal distribution.

### 6.3 Using sequence divergence to estimate RI

There is a plethora of methods for estimating rates of gene flow from genetic data, either applied to regions of genome or to the genome as a whole. However, RI is defined as the reduction in gene flow due to genetic differences. Therefore, to estimate RI, it would be necessary to obtain measures of both *m*_e_ (either along the genome or for an unlinked neutral locus) and *m*.

Wright (1950) introduced the classic statistic, *F*_ST_, which measures allele frequency differences between populations. Assuming an infinite island model, Wright suggested using *F*_ST_ to estimate the number of migrants between demes, $N_{e}m$, at an equilibrium between drift and gene flow, $F_{\text{ST}}=1/\left(1+4N_{e}m\right)$. When applied to empirical data, *F*_ST_ reflects the migration actually experienced by the loci analysed (rather than the raw migration rate), and thus in most cases would more accurately be described as $F_{\text{ST}}=1/\left(1+4N_{e}m_{e}\right)$.

Partly for that reason, *F*_ST_ varies along the genome, and a main premise of speciation genomics has been that genomic regions containing barrier loci (‘genomic islands of divergence’) can be discovered by ‘scanning’ genomes for high‐*F*_ST_ windows (Ravinet et al., 2017). *F*_ST_‐based genome scans have now been applied to countless systems and, in some cases, have revealed regions where gene flow appears to have been locally reduced.

However, interpreting *F*_ST_ in terms of gene flow is difficult (Whitlock & McCauley, 1999). *F*_ST_ is a relative measure, which can be inflated by reductions in genetic diversity due to selective sweeps or background selection, independent of gene flow. Indeed, in some systems ‘islands’ appear to have been shaped by these processes (Burri et al., 2015; Chase et al., 2021; Cruickshank & Hahn, 2014). There is also wide variation in *F*_ST_ even in the absence of such causes, due to the fundamental randomness of the evolutionary process, making it difficult to reliably detect local barriers in the genome (Lohse, 2017). Despite these difficulties, *F*_ST_ can be a useful indicator of divergence, but can only be taken as evidence for reduced effective gene flow after correction for confounding factors and when combined with other evidence, e.g. from experiments (Ravinet et al., 2017).

In an alternative form of genome scan, some studies have used the admixture proportion, *f*_d_, to characterize patterns of introgression across genomes (Martin et al., 2019; Ravinet et al., 2021; Stankowski et al., 2019). Like other *D*‐statistics (Green et al., 2010), *f*_d_ measures introgression from the excess of shared derived sites in a four‐taxon framework, but is modified for application to genomic windows (Martin et al., 2015). The underlying assumption is that ‘ABBA’ and ‘BABA’ site patterns should be equally frequent in the genome when sharing of the derived allele (B) results from random sorting or recurrent mutations. Gene flow, on the other hand, creates an excess of one site pattern, which can be used to identify and quantify introgression.

Estimates of *f*_d_ are roughly proportional to admixture for small simulated genomic windows (Martin et al., 2015), so one would expect scans of *f*_d_ to be correlated with variation in *m*_e_ across the genome (Martin et al., 2015, 2019). Also, unlike *F*_ST_, *f*_d_ is robust to variation in nucleotide diversity across the genome, so should largely be unaffected by sweeps or background selection. However, *f*_d_ is slightly biased towards regions with low between‐population divergence (low *d_xy_* or shallow between‐species coalescence), so scans with multiple statistics may give a clearer picture (i.e. *F*_ST_ and *f*_d_).

Alternatives to genome scans circumvent some of the problems listed above. For example, Aeschbacher et al. (2017) use a genome‐wide pattern rather than focussing on small windows, potentially increasing power. They use the genome‐wide negative correlation between recombination rate and population divergence found under highly polygenic RI, which appears because higher recombination rates allow neutral loci to decouple from divergently selected loci and hence introgress. Using coalescent theory, Aeschbacher et al. (2017) fit a model of divergence with gene flow to empirical divergence data and generate estimates for the selection density (the product of the mean selection coefficient and the density of selected sites) and the baseline migration rate (*m*). Because they specify how *m*_e_ is determined by the selection density per map length, *r* and *m*, it is possible to calculate *m*_e_, and therefore RI, as a function of the recombination rate. This method thus estimates how *m*_e_ generally depends on the recombination rate in the focal system, but it does not actually directly give measures of local RI along the genome. This approach aggregates information across the whole genome and does include a correction for background selection, but nevertheless depends strongly on the demographic model and on the assumed spatial structure.

Various methods for modelling demographic history (including gene flow) have become popular, e.g. those based on the site frequency spectrum (dadi—Gutenkunst et al., 2010; fastsimcoal2—Excoffier et al., 2021) or summary statistics in an ABC framework (Beaumont et al., 2002; Fraïsse et al., 2021; Roux et al., 2016). These approaches do not necessarily rely on equilibrium or infinite islands assumptions. Focussing on putatively neutral markers and neutral demographic processes, they are often treated as fundamentally separate from genome scan methods (focussed on finding selected loci). Demographic modelling has been used to obtain estimates of *m*, but some recent approaches have also explicitly considered the fact that not all loci are neutral, and fit a distribution of *m*_e_ rather than a single value while also taking other potentially confounding processes into account (e.g. pervasive background selection) (Fraïsse et al., 2021; Rougemont et al., 2017). Emerging methods aim to explicitly characterize variation in *m*_e_ across the genome by fitting separate demographic models to defined blocks of sequence (Laetsch et al., n.d.). However, like genome scans, these approaches try to estimate gene flow from genomic data affected by various processes, and failing to include those in the model might lead to serious errors when estimating gene flow (e.g. Momigliano et al., 2021).

Importantly, all approaches listed in this section suffer from the same fundamental problem: They do not generate the estimate of *m* needed to calculate RI. Even though some methods aim to estimate *m*, they instead estimate *m*_e_, the gene flow actually experienced by the loci analysed. We have seen above that most RI may be due to the aggregate genome‐wide effect of divergent selection, i.e. a general barrier to gene flow that affects the whole genome. This means that any estimate of gene flow obtained from genomic data, even from a genomic region distant from any strongly selected locus, includes the effect of this genome‐wide barrier, and is thus an estimate of *m*_e_ that is lower than *m*. Genome scans alone, for example, can therefore potentially identify genomic regions where *m*_e_ is reduced *on top of the effect of the genome*‐*wide barrier to gene flow*, but they cannot be used to find *m* and so generate an RI estimate that is comparable among different systems. Similarly, demographic modelling that aims to estimate *m* cannot distinguish to what extent a limitation in gene flow is due to physical versus genetic barriers.

Here, it becomes important to combine genomic data with data closer to those obtained by the ‘organismal’ methods described above. For example, migration rates can be estimated directly with mark‐recapture experiments (MacCallum et al., 1998) or using observations of dispersal in pedigreed populations (Aguillon et al., 2017). However, it needs to be noted that these organismal methods reflect *m* on the timescale of a few generations, while genomic estimates of *m*_e_ from e.g. IM model fitting reflect long‐term gene flow over thousands of generations. Therefore, in non‐equilibrium scenarios the combination of these estimates for *m* and *m*_e_ cannot lead to reliable estimates of RI.

A final point to note for the methods covered in this section is that these approaches (except, maybe, for demographic analyses explicitly taking into account hybrid zone settings) should be used with caution when the study system forms a hybrid zone rather than more or less discrete populations. As we have discussed above, reproductive barriers play out differently in continuous space; *m*_e_ has no clear definition in continuous space, and assumptions of the models underlying two‐deme approaches may be violated.

### 6.4 Conclusions about estimating RI from empirical data

Estimating RI from empirical data is extremely challenging. Methods determining short‐term RI based on the fitnesses of migrants, hybrids and backcrosses in the field might be most promising for spatially discrete populations or sympatric taxa with low levels of gene flow (Martin & Wainwright, 2013; McBride & Singer, 2010; Schluter et al., 2021). These approaches are limited to estimating genome‐wide RI. However, they could be extended to understand local RI by observing how blocks of introgression are distributed across the genome (e.g. Petr et al., 2019): In genomic regions with higher RI, introgression is expected to be reduced. However, testing whether there is significant variation in introgression is challenging, since the process is highly random.

In continuous space, hybrid zone analysis is promising if there is detailed sampling of spatial clines. Genomic data used in e.g. genome scans certainly often reflect RI to some extent, but a major challenge here is to disentangle *m* and *m*_e_. While non‐genetic data on migration rates (e.g. mark‐recapture experiments) might be useful to some extent, they reflect *m* over much shorter timescales than the genomic data.

RI estimators based on mating experiments or crosses are unlikely to reflect RI as defined here. For example, experimental estimates of reproductive barriers made in one or a few generations may be high, but these barriers may not be very isolating in the natural setting, or over timescales that are more relevant to gene flow. However, experiments with organisms are necessary to determine the barriers that reduce gene flow, and careful observations of morphology and behaviour are often necessary to define the groups between which to measure RI in the first place.

## 7 WHY SHOULD WE CARE ABOUT RI?

Reproductive isolation has received much attention because of its central importance to the biological species concept. We define RI in terms of the effect of genetic differences on gene flow. We define RI only for neutral loci in order to separate it from the idiosyncratic effects of selection on particular alleles. However, in most studies of adaptation and speciation, interest focuses primarily on traits and loci under selection. Why should we care about a quantity that is only defined for neutral alleles?

RI as defined in this article compares the actual migration rate, *m*, and the effective migration rate, *m*_e_. Knowing *m*_e_ for an unlinked neutral locus is useful in itself, since it estimates the realized background rate of gene flow, which is relevant not only for neutral, but also for selected loci. Local adaptation can be maintained if divergent selection is stronger than the effective migration rate into a deme, or if the area under divergent selection is sufficiently large, and the barrier sufficiently strong (Piálek & Barton, 1997; Turelli & Barton, 2017). The role of a reduced effective migration rate (due to a genetically based genome‐wide barrier) for the accumulation of divergence at further small‐effect loci has been highlighted in the discussion about ‘genomic hitchhiking’ (e.g. Flaxman et al., 2013). Knowing *m*_e_ may also be important for predicting the spread of universally favoured alleles, e.g. herbicide resistance between different populations or species of weeds. However, favourable alleles will relatively quickly penetrate all but the very strongest barriers (See Appendix S2, Consequences of barriers, for a detailed summary).

Thus, *m*_e_ is useful and sufficient if we want to predict how neutral, locally or universally adaptive alleles flow between different demes on the short term. However, in speciation research we are not only interested in how and to what extent gene flow is limited between groups of individuals, but in the extent to which this limitation is *caused by inherent differences between these groups*, and in how such differences allow them to coexist in sympatry without collapsing. To measure this intrinsic component specifically, *m*_e_ is not adequate, as it reflects both limits to the baseline migration rate and the effects of genetic barriers to gene flow. We need both *m* and *m*_e_, as only the ratio between the two is caused by genetic differences. This is why we need to measure RI. We can then relate RI to other features of the system to better understand the processes and barriers that contribute to speciation.

For example, some organismal approaches measure the extent of assortative mating. To understand whether and how mate choice contributes to speciation, we then need to ask: Does assortative mating substantially reduce gene flow? To answer that question, we need to determine RI with and without assortative mating (which could be done in experimental populations or using simulations), and we also need to know the genetic basis of assortment. On the other hand, if we observe strong RI for some taxon pair, we can ask: which barriers to gene flow do we find on the organismal level, and are these barriers sufficient to explain such a high level of RI?

This idea of using measures of RI to understand the speciation process is represented in the concept of a ‘speciation continuum’. This concept relies on using contemporary population pairs, varying in their level of RI, to reconstruct the speciation process (Stankowski & Ravinet, 2021a). As Stankowski and Ravinet (2021a) point out, this approach may be flawed because different contemporary taxon pairs may have followed very different evolutionary trajectories, not representing the same single speciation process. However, comparative analyses can allow us to identify different factors that vary with, and potentially cause, variation in the strength of RI. In the present article, it also becomes clear that the comparative measures of RI necessary for this approach may be difficult to obtain. Most genomic measures of RI will be influenced by differences in the history and spatial situation of the individual taxon pairs. Again, field studies across multiple hybrid generations and hybrid zone analysis might be the most promising ways to infer RI.

RI, even if reliably measured, is not sufficient to predict coexistence in sympatry. While some RI is necessary for maintaining sets of adaptive alleles without being broken up by recombination, long‐term full sympatry requires ecological divergence. Even if hybrids are completely inviable, cross‐mating will be more costly to the rarer population and may prevent coexistence. Here, it again becomes apparent that a focus solely on genetic patterns and processes is not sufficient, and that ecological processes need to be considered to comprehensively understand speciation.

## 8 CONCLUSIONS

This article is our attempt to clarify several key issues surrounding reproductive isolation, including what RI is, how it can be quantified in principle, and how it can be measured in practice. We define RI based on the reduction in gene flow between populations that is due to genetic differences. We have shown that RI depends strongly on circumstances, including the spatial, temporal and genomic context. This makes it difficult to quantify RI in a way that will be directly comparable across systems. After reviewing methods for estimating it from empirical data, we conclude that it is difficult to measure RI in practice. All existing methods have shortcomings and assumptions that will limit their applicability and accuracy.

A main issue is that existing definitions and measures of RI, including those we prefer, only apply in situations where most of the individuals can be assigned to one of two (or more) distinct populations, without the occurrence of complex hybrids across large spatial areas. In some taxa, hybridization is pervasive and it is impossible to identify distinct groups between which RI can be measured. In such cases, it is unclear what we would want to measure in the first place. Future work should develop concepts and measures for such scenarios.

While these messages may seem overly negative, we emphasize that in many systems RI is useful and necessary for quantifying the evolutionary independence of populations. While not perfect, existing methods, especially when combined and interpreted with appropriate caution, can give insight into the extent to which populations evolve independently and the underlying barriers to gene flow. Looking to the future, we encourage researchers to explore new, creative approaches to estimating RI in the field, taking advantage of all of the available data and combining measures of RI with experiments and field data on the different contributing barriers.

## CONFLICT OF INTEREST

The authors declare no conflict of interest.

## AUTHOR CONTRIBUTIONS

AMW and SS conceived the idea of the article. NB provided new analytical results and PS conducted simulations. AMW, SS and NB wrote the article after discussions among all authors.

### PEER REVIEW

The peer review history for this article is available at https://publons.com/publon/10.1111/jeb.14005.

## Supporting information

Table S1

Table S2

Appendix S1

Appendix S2

## Data Availability

This work does not contain new data.
