# Supplementary material for: What is reproductive isolation?
Source: J Evol Biol. 2022 Sep 5;35(9):1143–64. doi: 10.1111/jeb.14005 (PMC9542822; doi:10.1111/jeb.14005)
Supplement: Supplementary file 1 — Table S1 [file JEB-35-1143-s004.doc]

**Table S1. Verbal definitions of RI given in selected publications**. All papers listed here use ‘reproductive isolation’, but do not always define it. If an explicit definition was not given, we consider that RI was not defined. We classified definitions as ‘organismal’ or ‘genetic’.

| Reference | Verbal definitions | Classification |
| --- | --- | --- |
| Emerson (1935) | Not defined |  |
| Mayr (1942) | Not defined | NA |
| Dhobzansky (1951) | “*The gene exchange between species is restricted or suppressed owing to genotypically conditioned differences between their populations”* pp. 181 | Genetic |
| Mayr (1959) | *“Reproductive isolation, however, refers to what we might call the protective devices of a well-integrated and harmoniously coadapted gene pool against pollution by other gene pools”* pp. 226 | Organismal |
| Mayr (1963) | Not defined |  |
| Felsenstein (1981) | Not defined | NA |
| Coyne & Orr (1989) | Not defined |  |
| Wu (2001) | Not defined | NA |
| Gavrilets (2004) | “*Reproductive isolation is defined as reduction or prevention of gene flow between populations by some differences between them*” pp. 9 | Genetic |
| Coyne & Orr (2004) | Not defined | NA |
| Price (2008) | Not defined |  |
| Sobel et al. (2010) | Not defined | NA |
| Nosil 2012 | Not defined | NA |
| Butlin et al. 2012 | “*Reproductive isolation occurs when two populations produce fewer viable and fertile offspring than expected from their relative abundance in a locality*” pp.27 | Organismal |
| Sobel & Chen (2014) | Not defined | NA |
| Barraclough (2019) | “*The lack of interbreeding between two populations or species of sexual organisms*” pp. 9 | Organismal |
| Stankowski & Ravient (2021) | “*A reduction in potential gene flow between populations caused by isolating barriers*” pp. 1257 | Genetic |

Emerson, A. E. (1935). Termitophile distribution and quantitative characters as indicators of physiological speciation in British Guiana termites (Isoptera). *Annals of the Entomological Society of America*, 28(3), 369-395.

Mayr, E. (1942). *Systematics and the origin of species, from the viewpoint of a zoologist*. Harvard University Press.

Dobzhansky, T. (1951). *Genetics and the Origin of Species*. 3^rd^ edition: Columbia university press.

Mayr, E. (1959). Isolation as an evolutionary factor. *Proceedings of the American Philosophical Society*, 103(2), 221-230.

Mayr, E. (1967). Animal species and evolution. Harvard University Press.

Felsenstein, J. (1981). Skepticism towards Santa Rosalia, or why are there so few kinds of animals? *Evolution,* 124-138.

Coyne, J. A., & Orr, H. A. (1989). Patterns of speciation in Drosophila. *Evolution,* 43(2), 362-381.

Wu, C. I. (2001). The genic view of the process of speciation. Journal of evolutionary biology, 14(6), 851-865.

Gavrilets, S. (2004). *Fitness landscapes and the origin of species (MPB-41)*. Princeton University Press.

Coyne, J. A., & Orr, H. A. (2004). *Speciation*. Sunderland, MA: Sinauer Associates.

Price, T. (2008). *Speciation in birds*. Roberts and Co.

Sobel, J. M., Chen, G. F., Watt, L. R., & Schemske, D. W. (2010). The biology of speciation. *Evolution, 64*(2), 295-315.

Nosil, P. (2012). *Ecological speciation*. Oxford University Press.

Butlin, R., Debelle, A., Kerth, C., Snook, R. R., Beukeboom, L. W., Cajas, R. C., ... & Network, M. C. S. (2012). What do we need to know about speciation? *Trends in Ecology and Evolution*, *27*(1), 27-39.

Sobel, J. M., & Chen, G. F. (2014). Unification of methods for estimating the strength of reproductive isolation. *Evolution*, *68*(5), 1511-1522.

Barraclough, T. G. (2019). *The evolutionary biology of species*. Oxford University Press.

Stankowski, S., & Ravinet, M. (2021). Defining the speciation continuum. *Evolution*, *75*(6), 1256-1273.
