# Supplementary material for: What is reproductive isolation?
Source: J Evol Biol. 2022 Sep 5;35(9):1143–64. doi: 10.1111/jeb.14005 (PMC9542822; doi:10.1111/jeb.14005)
Supplement: Supplementary file 2 — Table S2 [file JEB-35-1143-s002.doc]

**Table S2. Researcher thoughts on the meaning of reproductive isolation revealed by an online survey.**  For details of the survey methodology, see Stankowski & Ravinet 2021. We focused on the answers of 230 respondents that answered ‘*Yes*’ to the question ‘*Do you study speciation*?’ and that provided a written answer to the question ‘*In a sentence or two, what is reproductive isolation?’*. We classified answers based on whether they had an (O)rganismal, (G)enetic, (D)istinctness, or other focus, or some combination of foci (e.g., O,G), as described in Box 1.

| **Focus** | **Answer to: “*In a sentence or two, what is reproductive isolation?”*** |
| --- | --- |
| D | The ability to coexist in sympatry in the field |
| D | The phenomenon that when populations that could potentially intergrade when in contact do not do so (or would not do so, if currently not in contact). |
| D | When two species hybrids are not detected in wild populations they are reproductively isolated. |
| D | the maintenance of independent evolutionary lines despite some (or no) ongoing interbreeding leads to a functional separation. |
| D | Coexistence in sympatry |
| D | Differentiation between two groups of individuals by pre- or postzygotic means |
| D | Any process that permits two groups to persist as distinct in sympatry |
| D | A mechanism that isolates two groups of individuals sufficiently strongly that linkage disequilibrium is maintained among many genetically unlinked loci in sympatry |
| D | The formation of new species which are distinct from their ancestral lineages due to evolutionary processes. |
| D | The situation when offspring from the parental populations in questions are not found in nature. |
| D | Reproductive isolation is meaningful in the field of speciation research when it can be demonstrated that free admixture of the genomes does(did) not occur when it has(had) an opportunity to do (have done) so. |
| D | Several mechanisms that maintain species apart |
| D | Emergence of discontinuity |
| G | A reduction in gene flow between populations below the within-population expectation |
| G | The disruption of gene flow between two lineages which most often share their MRCA. |
| G | The reduction of gene flow between populations. |
| G | Barriers to gene flow at any stage (e.g. mating, gametic, postzygotic) |
| G | The development of incompatibilities, or other barriers to gene flow, so that two lineages can remain isolated even when they overlap in distribution. |
| G | Greatly reduced genetic exchange between populations' genomes |
| G | Cessation of gene flow between populations |
| G | Barriers to gene flow, mechanism at the phenotypic or Ecological level that create statistical LD by preventing overall alleles mixing better two groups |
| G | Genetic differences that prevent flow of genes between populations |
| G | a small likelihood of gene flow |
| G | Mechanisms that prohibit two populations from having substantial gene flow. These mechanisms might be geographical or the result of differentiation between populations. |
| G | Restriction of genetic exchange |
| G | Lack of gene flow between species |
| G | Reduction of gene flow between populations due to biotic rather than abiotic factors. |
| G | lack of gene flow between populations |
| G | A description of (i.e. there is no purpose) of the reduction of gene flow between two taxa or populations. In can increase or decrease over time, it can vary among populations and across the genome. |
| G | The evolution or creation of biological of physical barriers to gene flow. |
| G | The average number of generations a randomly selected introgressed allele will persist in the population is less than, say, 5. |
| G | No, or very limited gene flow between groups over the complete genome |
| G | The absence of gene flow between particular populations long enough to allow for appreciable divergence. |
| G | Reproductive isolation refers to the absence of effective gene flow between populations. |
| G | Any barrier reducing gene flow between populations /species, by reducing mating/fertilization success or affecting hybrid fitness |
| G | When there is no gene flow among populations |
| G | Gene flow is absent, or almost absent, and is too low to maintain/prevent divergence |
| G | Cessation of exchange of genetic material over a period of time leading to diagnosable character differences |
| G | An absence of gene flow between populations. |
| G | The cessation of gene flow between groups of individuals. |
| G | Limited genetic exchange between two populations over time |
| G | when two sympatric populations are not exchanging genetic material; if the populations in question are allopatric, when we have reason to believe that they would not exchange genetic material were they brought back into sympatry |
| G | Whatever reduces gene flow among lineages if and when they would meet |
| G | Cessation of gene flow. |
| G | When two (sub)populations cease to exchange genes. |
| G | geographical and genetic barriers or selective traits that prevent gene flow |
| G | absence of gene flow between populations |
| G | Limited gene flow between populations |
| G | Absence of gene interchange among populations |
| G | Reproductice isolation is the phenomenon that gene flow between different species becomes supressed. |
| G | Isolation of genomes |
| G | The absence of gene/migrant exchange between populations |
| G | reduced capacity to exchange genetic material |
| G | Factors that limits gene flow between populations. |
| G | Barriers acting to reduce to prevent gene flow. |
| G | Anything that reduces gene flow between two populations relative to expectations under random mating. |
| G | The lack of gene flow between populations |
| G | A reduction in gene flow between two populations. |
| G | Any mechanism that reduces effective migration from expectations of neutral panmixia between two groups in contact [if I could underline or bold it - I would stress that this "in contact" part is absolutely crucial] |
| G | Reduction in the exchange of genes with members of other populations. Can range in magnitude from low (partial RI) to high (100%; complete RI). |
| G | In essence, reproductive isolation is tokogenetic cessation. |
| G | Restricted gene flow between subgroups |
| G | zero or reduced gene flow among divergent lineages |
| G | barrier to gene flow between taxa |
| G | The incapacity for two groups of individual to exchange all or a part of their genes |
| G | Lack of gene flow between two populations. |
| G | RI means that there are barriers to gene flow between groups of individuals. |
| G | Lack of gene exchange between populations |
| G | For corals this is difficult, they are known to hybridise. For my work, I try to determine demographic levels of geneflow (or lack of) as a measure of isolation or connectivity. |
| G | a barrier to gene flow |
| G | Two populations are reproductively isolated when there is no gene flow between them anymore, and the barriers to gene flow are maintained over time |
| G | the establishment of barriers limiting or stopping gene flow between populations or groups |
| G | the accumulation of barriers to gene flow between groups/populations |
| G | lack of gene flow |
| G | A set of mechanisms that reduce gene flow among populations. |
| G | reduced gene flux in the absence of ecological/geographical barrier |
| G | The existence of barriers that impede or strongly limit gene flow between closely related species |
| G | Any kind of mechanism reducing gene flow between two population / species |
| G | the mechanisms preventing gene flow |
| G | Populations actually not exchanging genetic material for some time |
| G | Lack of gene flow |
| G | It is a process by which exchanges of genetic material became less and less frequent. |
| G | Lack of gene flow between two lineages |
| G,D | The reduction in gene flow among population which allows a population to maintain phenotypic and genetic cohesiveness |
| G,D | A barrier to gene flow between two distinct taxa. |
| O | offspring produced by interbreeding are less fit |
| O | Reproductive isolation describes the situation where individuals drawn from two different populations are less successful at producing viable, fertile offspring than individuals drawn from the same population. It is non-binary. |
| O | The presence of barriers to reproduction between lineages |
| O | Nearly complete cessation of successful interbreeding between two differentiated lineages upon secondary contact. |
| O | Reproductive isolation is an umbrella term encapsulating any isolating mechanism/difference (intrinsic or extrinsic) that prevents two groups from producing viable, fertile offspring. |
| O | Any reduction in offspring fitness between two (genetically identifyable) groups of organisms |
| O | Reduction in the probability of producing viable and fertile offspring relative to an expectation based on spatial separation alone. |
| O | Behavioral, geographic, ecological or other barriers between species that prevent interbreeding |
| O | RI occurs whenever two lineages (populations or whatever arbitrary group of individuals you want to call a lineage) are not randomly mating for some reason. |
| O | Mating barrier of some sort, pre or post mating. |
| O | A reduction in % of interbreeding success b/w groups relative to w/in group mating events. Doesn't have to prevent offspring formation fully - just enough to reinforce the evolution of prezygotic isolation when those two groups reunite in sympatry. Can be pre- or post-zygotic, or a combination of the two. |
| O | When two groups have the opportunity to reproduce but cannot or do not |
| O | Not producing fertile offspring |
| O | Reproductive isolations is any process that makes a zygote not viable |
| O | Physical, ecological, or behavioral barriers that prevent evolutionary lineages from merging. |
| O | The inability of lineages to produce reproductively viable offspring. |
| O | When two populations cannot interbreed via physical or biological distance |
| O | Inability to produce viable and/or fertile offspring (either partially or completely) |
| O | Reproductive isolation is any barrier to interbreeding between populations. |
| O | Populations would not interbreed in nature even if sympatric. |
| O | a general inability to produce viable and fertile hybrids in nature for reasons other than allopatry per se |
| O | The inability to produce viable offspring in a natural context. |
| O | Working on sympatric speciation, it‚Äôs sterility of hybrids. |
| O | Inability of two lineages to mate or the reduction in hybrid fitness when two lineages mate with each other. |
| O | Assortative mating |
| O | Selection against interbreeding acting at any level, to the point where hybrids are rare or in a narrow zone. Cannot be directly assessed in island populations, but can be inferred by comparable terrestrial systems. |
| O | Physical barriers/isolation and/or genetic incompatibilities which blocks organisms to mate or cause reduced offspring viability etc. |
| O | A behavioral mechanism, trait or genetic incompatibility that reduces interbreeding and hybrids |
| O | Reduced sexual compatibility between populations |
| O | The inability to produce a viable offspring whether that be through fertilization barriers, genetic/genomic incompatibilities or different pollination ecology. |
| O | The lack of offspring that is capable of producing offspring - can be pre- or postzygotic |
| O | The innate inability to exchange gametes between two or more gene pools. |
| O | The fact that two groups of similar organisms can not reproduce or produce fertile offspring |
| O | Any deviation from random mating |
| O | reproductive isolation is present when two populations cannot interbreed any more (to a large extent), due to any pre- or postzygotic barrier, which may be ecological, physiological, mechanical, genetic, or behavioral |
| O | a reduction in the frequency with which certain groups of individuals produce offspring with one-another |
| O | Offspring are less fit as hybrids than as non-hybrids. This ignores hybrid vigor. |
| O | Two sympatric populations are reproductively isolated when they do not interbreed and form viable offspring. Reproductive isolation can be pre-zygotic (e.g. no mating occurs for behavioural reasons) or post zygotic (mating occurs but viable offspring are not produced). |
| O | Reproductive isolation is realized when the chance of two organisms from different populations (or taxa) exchanging gametes is extremely low in realistic ecological conditions (as compared to the magnitude of the chance of exchanging gametes within the same population/taxon). |
| O | When most individuals of two diverged lineages cannot mate, either by producing inviable offspring or by avoid mating with individuals of the other lineage. |
| O | the stuff that prevents two groups of organisms successfully interbreeding when they share the same location |
| O | Separation into 2 groups that no longer freely interbreed and/or produce fit offspring |
| O | The accumulation of geographical, behavioral, and physiological (including genetic) barriers that prevent 2 populations/species from creating fertile hybrid offspring. |
| O | The things that limit or prevent reproduction between populations. |
| O | Any barrier (pre- or postzygotic) to the production of viable, fertile offspring. Should be measured relative to intraspecific (or intrapopulation) production of viable, fertile offspring. |
| O | the inability of a species (or incipient species/subspecies/population) to breed (i.e. mate or produce viable/fertile offsprings) successfully with related species (or populations etc...) due to geographical, ecological, behavioral, physiological, mecanical or genetic (not exhaustive list!) barriers |
| O | Reproductive isolation is a mechanism preventing the reproduction between two organism. Two organisms are productively isolated if they can not produce viable offspring and no gene flow occurs between them. |
| O | A mechanism that decreases the frequency or fitness of hybrids |
| O | Inability of two populations to interbreed and produce viable offspring |
| O | Reproductive isolation is the inability of two taxa to produce progeny. This may be from the inability to mate, the inability of gametes to fuse, or the inability to produce fertile offspring. |
| O | Reduced or precluded mating/fertilisation among members of different populations in relation to mating/fertilisation among members of the same population. |
| O | A relational concept arising from the evolution of traits that limit reproduction between two groups of individuals. |
| O | Infrequent ability to successfully produce fertile offspring |
| O | Reproductive isolation results from pre- and/or post-zygotic processes reducing the fitness of the parents due to genetic incompatibilities (in a broad sense) or maladaptations to the local environment following divergence. |
| O | Two groups of organisms are repoductively isolated from each other when a biological pre- or post-zygotic mechanism is established that prevents the production of viable progeny even when they co-occur. |
| O | Reduced capacity for interbreeding |
| O | Accumulation of barriers (e.g., ecological, mechanical, genetic) to gene flow between potentially interbreeding populations |
| O | Mechanisms that prevent related groups from successfully mating and producing viable offspring |
| O | Inability of a species to breed successfully |
| O | Reproductive isolation is the reduction of reproduction (or its correlates) between members of different species compared to that seen between individuals of the same species. |
| O | When divergence/drift has resulted in the inability of closely related species to successfully interbreed. |
| O | Failure to produce F2s in nature |
| O | The inability of organisms to interbreed due to genomic differences manifesting as behavioural or physiological barriers |
| O | When lineages are no longer capable of producing viable offspring following a breeding event. |
| O | Lower fitness of interspecific crosses as compared to intraspecific crosses |
| O | when 2 populations are not interbreeding |
| O | A reduction in the number of progeny produced during inter-group, relative to intra-group, mating. |
| O | When applied to two populations, the inability to generate fertile offspring from mating of individuals between populations. |
| O | Reduced fitness in the progeny of two or more populations due to intrinsic or extrinsic factors |
| O | The incapacity of two or more organisms to produce offspring through sexual reproduction, due to biological, behavioral and environmental factors (and maybe more!). |
| O | any barrier to the formation of viable and fertile hybrids |
| O | When there are physical and/or pre POS zigotic barriers |
| O | Individuals from different populations successfully reproduce less than within population pairs: Perhaps the populations never meet; perhaps offspring are genetic dead-ends. |
| O | When breeding among different species or populations is not possible. |
| O | Reproductive isolation is the complete (not just partial) inability of two organisms of different sex (i.e. male and female) to produce offspring that are, themselves, able to sexually reproduce |
| O | external or intrinsic barrier to reproduction |
| O | Offspring betw. parental species is not fertile, or some later generation hybrids are not fertile. |
| O | a deviation from random mating |
| O | Inability to produce viable or fertile offspring after max 2-3 generations of hybridisation |
| O | a barrier to successful mating |
| O | Absence of fertile decent |
| O | When two or more populations cannot interbreed due to a variety of factors, predominantly genetic incompatibilities (affecting viability and fertility) and mating preferences. |
| O | Reproductive isolation is when two species have barriers to being able to interbreed. |
| O | no ability to produce fertile offspring between two populations/groups. |
| O | barriers that prevent species from mating, prevent the formation of hybrids, or cause hybrid inviability and/or sterility |
| O | The inability of two individuals to interbreed or to interbreed and give rise to fertile offspring |
| O | Any form of barrier that prevents two populations from interbreeding |
| O | If individuals from two populations do not (or only rarely) mate with each other. |
| O | Barriers in producing viable or fertile progenies |
| O | Absence of inter-breeding among populations due to pre- or post-mating mechanisms |
| O | Geogaphical - habitat - in plants pollination/fertilation mechanisms |
| O | When individuals of one population do not breed successfully with individuals from another population in sympatry. At the very least the progeny of two individuals from two different populations have lower fitness than if they bred within their own populations. |
| O | The process of accumulation of reproductive isolation barriers over time leading to complete reproductive isolation. |
| O | They do not recognize each other as a partner of reproductive behavior |
| O | Biological characters, those prevent inter-population cross |
| O | Reproductive isolation is a condition where two species cannot produce a long-lasting population of hybrid offspring, and the barrier leading to reproductive isolation can be pre- or post-zygotic. |
| O,D | The combination of all extrinsic and intrinsic barriers that prevent species from merging together in sympatry. |
| O,D | Reproductive isolation is a collection of pre- or postzygotic mechanisms that prevents two species from merging into one. |
| O,G | Mechanisms that reduce gene flow between populations, ecotypes, or species. |
| O,G | The multivariate assembly of mechanisms that prevent interspecific gene flow. |
| O,G | The diminishment of gene flow between two sets of populations, modulated by a trait or set of traits that appear either in the parental populations themselves or in their hybrids. |
| O,G | the extent to which populations do not exchange genetic material through interbreeding |
| O,G | A heritable trait that results in a reduction of potential gene flow |
| O,G | RI is often used to describe the influence of trait differences on gene flow. |
| O,G | Groups of individuals that have pre- or post mating mechanisms that restrict the exchange of genetic material between them |
| O,G | From an individual point of view: The inability (or "unwillingness") to produce reproductively viable offspring. From a population point of view: The inability of two gene pools to exchange alleles. |
| O,G | Barrier to gene exchange due to pre- and post-zygotic, intrinsic and extrinsic mechanisms (everything that is not natural barriers to dispersal). |
| O,G | Reproductive isolation (RI) is the inability to produce successful offspring, applied at the level of a pair of reproductively mature individuals, or at the population level. RI maybe partial or complete. |
| O,G | A phrase that biologists use but actually mean very different things by. ;) I suppose it should mean any mechanism that reduces gene flow between two sexually-reproducing groups (either lack of interbreeding or low fitness of hybrids). |
| O,G | RI is a set of barriers, both prezygotic and postzygotic, that prevent substantial gene flow between two populations/species. |
| O,G | The restriction of gene sharing either by pre or post mating inhibition. |
| O,G | Reduction of actual or potential gene flow between taxonomic groups due to their biological properties, |
| O,G | a state in which groups of organisms are separated by barriers to gene flow (i.e. reproductive barriers) |
| O,G | Absence of gene flow between populations due to prezygotic or postzygotic barriers |
| O,G | When two species are suitably dissimilar or so geographically isolated from each other that they will no longer be able to exchange genetic material |
| O,G | Reproductive isolation is when groups of individuals reproduce within rather than between groups given the opportunity to do so (sympatry). This corresponds to a between population migration rate smaller than 0.5 among sympatric groups of individuals (=populations). |
| O,G | Reproductive isolation is the result of a set of mechanisms which impede genetic exchange between populations that were able to freely exchange in the past. |
| O,G | mechanisms that prevent gene flow with other potential partners |
| O,G | Exogenous and/or endogenous factors limiting the gene flow between diverging species and eroding the hybrids fitness |
| O,G | The evolution of biological mechanisms that prevent gene flow between lineages. |
| O,G | Reproductive isolation occurs when two taxa are unable to exchange genes due to intrinsic barriers. |
| O,G | Lack of gene flow due to interbreeding |
| O,G | Failure of two or more populations to interbreed and exchange genetic material. |
| O,G | The absence of gene flow between divergent populations/lineages/species due to mechanisms (pre- and/or postzygotic) that prevent interbreeding and or the production of viable/fertile offspring. |
| O,G | Reduction of gene flow between groups due to evolutionary changes in characters that affect mating (sensu latto, e.g. including changes in habitat preferences) or due to occupation of different geographic areas between which no gene flow can occur. |
| O,G | A sieving process that limits the exchange of genes (in perpetuity), be it ecological, behavioural, pre or postygotic. |
| O,G | evolution of barriers to reproduction to impede or prevent genetic exchange between individuals of incipient species |
| O,G | Curbed inter-population gene flow caused by reproductive barriers |
| O,G | When a mechanism or process hinders gene flow from from one population (species) to another, either by preventing interbreeding or by producing hybrids with low fitness. |
| O,G | The inability of members of two sexually reproducing species to exchange genetic material that results in viable and fertile offspring. |
| O,G | Reproductive isolation is the prevention of gene flow between diverging lineages, for example, limited distribution range overlap, ecological divergence, assortative mating or hybrid dysfunction. |
| O,G | Reproductive isolation is the reduction in gene flow between populations that results from isolating barriers. Isolating barriers are diverse: they may result from differences in ecology, behavior, genomics..., and act at different times (e.g. pre- or post-zygotic barriers, first- or later generations). |
| O,G | Reproductive isolation is the set of mechanisms that impede the exchange of genes between populations allowing genetic and phenotypic differences to accumulate, either by preventing mating or fertilisation, or resulting in low fitness hybrid offspring (including inviability/infertility). |
| O,G | mechanism that prevents gene flow between individual and/or populations |
| O,G | The existence of barriers to gene flow between population/species. These can be endogeneous (indepedent of environment) or exogeneous (dependent of envt) and prezygotic or postzygotic |
| O,G | The existence of pre-mating or postmating-mating barriers to gene flow. |
| O,G,D | any intrinsic or extrinsic feature that reduces gene flow between two genetically / phenotypically distinct clusters |
| other | The main (only?) focus of speciation research for generations that has created an obstacle for speciation research when other important evolutionary forces are also important (see Templeton 1989, Harvey et al. 2019) |
| other | Ha |
| other | Too complex to write in a sentence or two. |
| other | Precisely what "breaks" the species concept used. |
| other | Reproductively isolated populations |
| other | barriers including geographic or genetic |
| other | Others use RI to mean any process, such as selection, or mating behaviour that tends to limit gene flow between population, except geography. I don't think it's a very useful term, as it supposedly applies to whole organisms (rather than parts of the genome), and doesn't actually prevent gene flow or introgression over time, and it's liable to be mis-used to make it seem as a special kind of selection occurring only at the species level. |
| other | with enough reproductive isolation populations will become genetically diverged, eg > 10% average Fst. |
