# Supplementary material for: What is reproductive isolation?
Source: J Evol Biol. 2022 Sep 5;35(9):1143–64. doi: 10.1111/jeb.14005 (PMC9542822; doi:10.1111/jeb.14005)
Supplement: Supplementary file 3 — Appendix S1 [file JEB-35-1143-s001.doc]

**What is reproductive isolation?**

Simulation results

*Flow into a single deme*

We simulate a mainland-island model considering a diploid population with either two (one selected and one neutral) or three (two selected and one neutral) loci and two alleles (namely 1 or 2) at each locus. We assume that the haplotype 11 (or 111) is fixed in the island and the alternative haplotype 22 (or 222) is initially fixed in the mainland. Every generation a small fraction of migrants enter the island from the mainland with rate
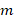
. The selection coefficient,
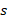
, is constant for each selected locus, with the diploid genotype fitnesses following heterozygote disadvantage in the ratio
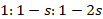
 and with multiplicative fitness. The population is simulated until an equilibrium is reached. The haplotype frequencies are followed and the strength of reproductive isolation (RI) is calculated for the neutral locus. We also consider different recombination rates,
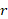
, between the neutral and selected loci, so as to calculate how the strength of RI varies along the genome.

A specific example is shown first, with one selected and one neutral locus with
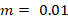
,
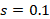
 and
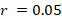
. Fig. S1.1 shows the allele frequency of the selected and neutral locus as a function of time (on a log scale). The allele frequency at the neutral locus shows a steady state decrease, the slope of which gives the effective migration rate (
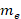
). This is found to be -0.0031. Since the allele frequency decreases over time, the slope is negative, but we use only the absolute value to calculate RI as
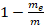
. Furthermore,
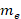
 is simply the allele frequency changes in a generation (
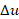
), relative to the allele frequency difference between demes (
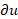
). A plot of
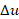
 vs.
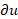
 gives a straight line, suggesting that
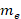
 is constant over time (see Fig. 5A in the main text).


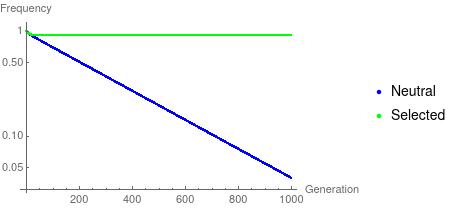


Fig. S1.1: Top: The allele frequency over time of the neutral (blue) and the selected (green) loci from a mainland-island model with
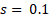
,
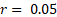
 and
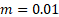
 plotted on a log scale. The rate of change of the allele frequency of the neutral locus is 0.0031.

Next, we simulate the population for different recombination rate between the neutral and selected loci with
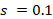
 and
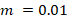
 throughout and calculate strength of RI for each case. The strength of RI is also calculated for the case with two selected and one neutral locus, again with
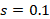
 for both loci and
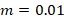
. We consider the neutral loci to be at either ends of the selected loci or between them to see how RI varies along the genome. We also verify the results from the simulation to the analytical result of RI (see main text). These results are shown in Fig. 3 of the main paper.

*One-dimensional hybrid zone*

Here, we assume a 1D stepping stone model with 101 demes and nearest neighbour migration with rate
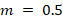
. The haplotype 11 (or 111) is fixed in the first 50 demes and the alternative haplotype 22 (or 222) is fixed in the remaining demes. At the selected site, the alternative alleles are selected in the first 50 and the last 51 demes. As before we assume heterozygote disadvantage and multiplicative fitness. For a simplified calculation of RI, we keep the haplotype frequencies always fixed for the first and last demes (111 is fixed in deme 1 and 222 in deme 101). The population is simulated until neutral allele shows a stable pattern.

Fig. S1.2 shows allele frequency over space for different generation for the case with
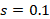
 and
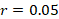
 between neutral and selected locus (in blue). It takes about 5000 to 10000 generations for the allele frequency of the neutral locus to stabilize. The barrier strength is then calculated as
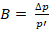
, where numerator is the step or the difference in the allele frequency between the tails and denominator is the rate of change of allele frequency in the tails. To calculate this from the simulations, we need to define a cut-off for the tails. We use demes 1 to 40 at one end and 61 to 101 at the other, and use regression to find their gradients, the mean of which gives
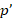
. The predicted allele frequencies at deme 50 is calculated from both tails, the difference between which gives
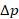
. A barrier strength of 8 is obtained for this case. As before, we now calculate the strength of the barrier,
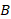
, for different positions of the neutral loci along the genome, with either one or two selected loci. This is shown in Fig. 4 in the main text.


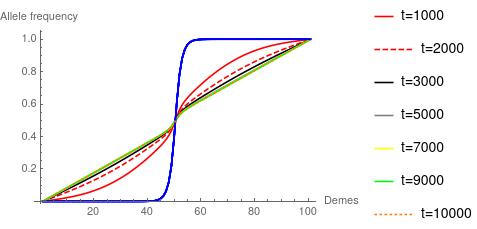


Fig. S1.2: Allele frequency of the neutral locus for different generations over space (top).

Next, the allele frequencies at the first and last deme is let to vary to represent a more plausible biological scenario. 201 demes are used instead of a 100 along with stronger selection of
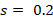
 and lower recombination rate (
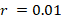
). The hybrid zone is simulated for 30000 generation. The allele frequency changes over space along with the associated barrier strength is shown in Fig. 5B in the main text.

*Hybrid zone with a physical barrier*

Here, we consider the 1D stepping stone model as before, with an additional assumption of a physical barrier between demes 50 and 51. The migration rate is
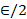
 between the demes, where
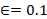
. As before, we calculate RI along the genome for the following cases

1. with just a physical barrier as mentioned above with no selection (i.e no genetic barrier) shown in grey

2. with only a genetic barrier (in black)

3. with both physical barrier and selection (in red)

4. when we add the effects of physical (1) and genetic barrier (2) (in blue)

and compare RI among them.


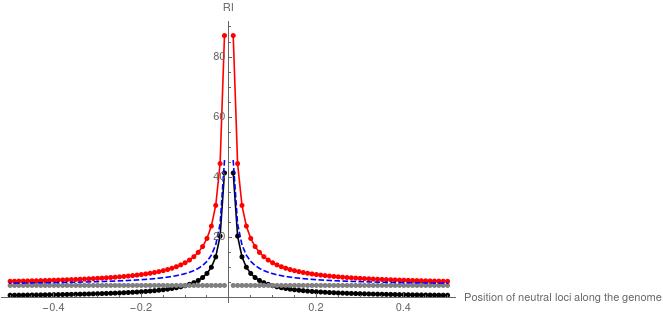


We see that the barrier strength with both physical and genetic barrier is greater than their effects combined.
